# Effects of blue light on flavonoid accumulation linked to the expression of miR393, miR394 and miR395 in longan embryogenic calli

**DOI:** 10.1371/journal.pone.0191444

**Published:** 2018-01-30

**Authors:** Hansheng Li, Yuling Lin, Xiaohui Chen, Yu Bai, Congqiao Wang, Xiaoping Xu, Yun Wang, Zhongxiong Lai

**Affiliations:** Institute of Horticultural Biotechnology, Fujian Agriculture and Forestry University, Fuzhou, Fujian, China; United States Department of Agriculture, UNITED STATES

## Abstract

While flavonoid metabolism’s regulation under light conditions by structural genes and transcription factors is understood, the roles of microRNAs (miRNAs) in this pathway have been rarely reported. In this paper, the accurate control of light was firstly enabled through the specially designed plant growth chamber which ensures consistency and accuracy of the cultivation of longan ECs and the repeatability of the experiments. Then, longan ECs were cultured in this chamber for 25 days. The change of growth rate of longan ECs was compared under different light qualities (dark, blue, green, white, green), intensities (16, 32, 64, 128, 256 μmol ·m^-2^ ·s^-1^), and durations (8 h, 12 h, 16 h, 20h, 24h). Results indicated that longan ECs had a high growth rate in the condition of blue or green light, at intensity ranged from 16 μmol·m^-2^·s^-1^ to 64 μmol·m^-2^·s^-1^, and duration from 8 h to 16 h. In addition, the contents of total flavonoids, rutin, and epicatechin were determined. Results indicated that flavonoid contents of longan ECs reached the highest value under blue light, at 32 μmol·m^-2^·s^-1^ and 12h/d. Blue light promoted the accumulation of epicatechin, but inhibited the synthesis of rutin. Finally, the expressions of flavonoid pathway genes, miRNAs and target genes were analyzed by qPCR. These results indicated that miR393 and its target gene *DlTIR1-3*, miR394 and its target gene *DlAlMT12*, and miR395 and its target gene *DlAPS1* had a negative regulating relationship under blue light in longan ECs. Furthermore, miR393, miR394, and miR395 acted on target genes, which negatively regulated flavonoid key genes *DlFLS* and positively regulated key genes *DlCHS*, *DlCHI*, *DlF3′H*, *DlDFR*, *DlLAR*, and finally affected the accumulation of flavonoids. The treatment of longan ECs under the blue light at the intensity of 32 μmol·m^-2^·s^-1^ for 12 h/d inhibited the expression of miR393, miR394 and miR395, which promoted the expression of target genes and the accumulation of flavonoids and epicatechin, but inhibited the synthesis of rutin.

## 1 Introduction

Longan (*Dimocarpus longan* Lour.), Sapindaceae, is a tropical/subtropical fruit plant, with high nutritional and medicinal values. The medicinal ingredients in longan are mainly secondary metabolites, including flavonoid substances [1], which have important physiological significances in plants. These compounds control auxin transport, ultraviolet (UV) protection and resistance functions [2]. In addition, flavonoids are biologically strong natural antioxidants, and have anti-cancer and anti-aging effects, as well as other medicinal benefits [2].

The synthesis of flavonoid metabolites in plants is affected by environmental stress [3,4], and light is an important environmental factor that affects cellular physiology. Light exposure leads to broad changes in the flavonoid biosynthesis pathway [5,6].

Research on the light-controlled molecular regulatory mechanisms involved in flavonoid metabolism has progressed [7]. The mechanisms mainly include two kinds of control genes: structural and regulatory. Light treatments can significantly improve the expression levels of litchi flavonoid structural genes, such as chalcone synthase (*CHS*), chalcone isomerase (*CHI*), flavanone 3-hydroxylase (*F3H*) and dihydroxy flavonol reductase (*DFR*) [8]. The flavonoid contents in plants is closely related to the transcription levels of the structural genes, while the v-myb avian myeloblastosis viral oncogene homolog (MYB) transcription factors can regulate the transcriptional expression levels of flavonoid structural genes [9]. In addition, a number of MYB transcription factors are regulated by the light [7]. The main structural and regulatory genes of flavonoid biosynthesis under different light conditions have been cloned in various plants [2,7]. The regulatory mechanisms of the main structural genes have been elucidated; however, these mechanisms are very complicated in the plant flavonoid biosynthesis pathways, which involve a wide variety of enzymes whose synthesis is also affected by other factors [2,7].

While flavonoid metabolism’s regulation under light conditions by structural genes and transcription factors is understood [7], the roles of microRNAs (miRNAs) in this pathway have been rarely reported. Moreover, their regulatory mechanisms are unclear. The miRNAs have emerged as significant regulators of gene expression in plants and have important regulatory functions in stress responses, development and epigenetic phenomena [10,11]. Some light-responsive miRNAs play important roles in plant stress responses. For example, in some plant species, miR166 is induced under heat, cold stress and UV-B radiation [12]. Here, miR398 was up-regulated by the environmental light conditions, suggesting that the miR398-mediated Cu/Zn superoxide dismutase regulation is involved in a number of responsive pathways [13]. The functional element analysis of longan miRNA promoters also revealed that miR393, miR394 and miR395 contain many light reaction-related components [14] and that they mediate light’s influence on plants. In addition, miRNAs may regulate the secondary metabolism of plants [15]. *Arabidopsis*’ miR393 can act on auxin response factors 1 and 9, and thus, regulate glucosinolate synthesis [16]. The negative regulation of anthocyanin biosynthesis by a miR156-targeted *squamosa promoter-binding protein-like transcription factor* (*SPL*) was described by Gou et al. [17]. The functional element analysis of longan miRNA promoters also found that miR393, miR394 and miR395 contained MYB-binding sites that were involved in flavonoid biosynthetic gene regulation [14]. Thus, we hypothesized that miR393, miR394 and miR395 play important roles in light-related pathways that affect flavonoid metabolism.

In most of the related literatures, the definitions and descriptions of light sources are quite inaccurate, and the illumination inside the growth chambers is generally uneven [18,19]. These problems were solved in our laboratory by improving the light-control technology. Our group has established a longan embryogenic callus (longan EC) culture system to study the influence of light treatments on flavonoid metabolism. This laid an important foundation for the study of the secondary metabolism of longan cells, and we determined it could be accurately regulated by a light source.

Here, we investigated longan ECs cultured using light-control technology. The growth status and flavonoid contents were observed and analyzed. To understand the regulatory mechanism behind miRNAs roles in light-affected flavonoid metabolism in longan ECs, we compared and analyzed their relationships, including miR393 and the target gene *transport inhibitor response protein 1* (*DlTIR1-3*), miR394 and the target gene *aluminum-activated malate transporter 12* (*DlALMT12*), miR395 and the target gene *ATP sulfurylase 1* (*DlAPS1*), and flavonoid biosynthesis related genes *DlCHS*, *DlCHI*, flavonol synthase (*DlFLS*), *DlF3′H*, *DlDFR* and leucoanthocyanidin reductase (*DlLAR*). These studies provided new insights into the molecular mechanisms that allow light to influence plant flavonoid metabolism.

## 2 Materials and methods

### 2.1 Equipment improvement and illumination uniformity analysis

Traditional plant growth chambers are often equipped with light sources of unknown or inexact wavelengths that distribute uneven illumination and light intensity levels. Illumination uniformity was achieved through the improvement of plant growth chambers (S1 Fig). The central wavelengths of red, green and blue light were 660, 515 and 457 nm, respectively (S2 Fig). Actual measurements revealed that the illumination uniformity was consistent at the same horizontal plane (S3 Fig). Light quality, light intensity and photoperiod in the chamber were controlled by an intelligent system (S1 Fig). This ensured consistent longan-EC culture conditions, as well as accurate and repeatable experiments.

### 2.2 Plant material and light treatments

Longan ECs were obtained using previously published methods [20,21]. Longan ECs were alternately kept on Murashige and Skoog (MS, Phytotechnology M519) medium (2% sucrose and 6 g/L agar, pH 5.8) supplemented with 4.5 μM 2,4-Dichlorophenoxyacetic acid (2,4-D) and MS medium supplemented with 4.5 μM 2,4-D, 2.3 μM kinetin and 5 mg/L AgNO3, and were subcultured every 20 d. Based on previous studies of the longan ECs’ flavonoids, the former medium was selected for use with the light treatments [22].

Longan ECs were put into plant growth chamber and control groups were incubated in dark. Three different kinds of treatments were investigated in the experiments. 1) Both intensity and photoperiod were fixed at 32 μmol·m^-2^·s^-1^ and 12h/d respectively with light qualities setting at blue (457 nm), green (515 nm), white, and red (660 nm); 2) Both light quality and photoperiod were fixed at blue and 12h/d respectively with light intensities setting at 16, 32, 64, 128, and 256 μmol·m^-2^·s^-1^; 3) Both light quality and intensity were fixed at blue and 32 μmol·m^-2^·s^-1^ respectively with photoperiods setting at 8, 12, 16, 20, and 24h.

Equal pieces (0.04 g) of longan ECs with diameters of 5–7 mm were placed onto separate bottles (240ml). There were 30 bottles used in each treatment, which lasted for 25 d at 25 ± 2°C with a relative humidity of 55%–60%. Each treatment was repeated for three times. After the treatments, all of the materials were stored at −80°C for later use.

The browned and polluted longan ECs were removed, and 25 bottles of longan ECs of good growth status were chosen. The fresh weights of the longan ECs were determined, and the growth rate of each bottle was calculated. Growth rate = (Final fresh weight–Starting fresh weight)/Starting fresh weight × 100%. Longan ECs were observed using an inverted electron microscope (Leica DM IL).

### 2.3 Flavonoid determination

The flavonoid contents were determined using the method of Li [23] with some modifications. Longan ECs were freeze-dried for 1 d using a lyophilizer (LGJ-25C), ground into fine powders, and extracted with 10 mL 60% ethanol. The extraction process lasted for 1 h at 60°C in an Ultrasonic Washing Device (KQ-200SPDE). The extracts were centrifuged at 10,000 g for 10 min at 20°C. The supernatant was collected into new tubes. Using the chromogenic reaction method, the flavonoid levels were measured using a DU 640 spectrophotometer.

### 2.4 Epicatechin extraction and UPLC-MS analyses

The epicatechin contents were determined using the method of Zuo [24] with some modifications. The extraction was carried out using 1.0 g fine longan EC powder dissolved in 10 mL methanol solution and extracted for 60 min at 60°C using an Ultrasonic Washing Device (KQ-200SPDE). The extracts were combined and filtered, and then evaporated to dryness in a rotary evaporator. The dried extracts were dissolved in 5 mL methanol, and all samples were filtered using 0.22-μm syringe filter.

The following chromatographic conditions were used. The mobile phase consisted of water containing 0.1% formic acid (A) and methanol (B). The composition of the mobile phase was 5%–25% (B) for 0–12 min, 25%–35% (B) for 12–15 min, and 35%–5% (B) for 15–18 min. The column temperature was set at 30°C. The injection volume was 5 μL, and the flow rate was 0.2 mL/min. All standards and samples were detected by UPLC (ACQUITY UPLC H-Class) at a wavelength of 280 nm.

### 2.5 Rutin extraction and UPLC-MS analyses

The rutin contents were determined using the method of You [25] with some modifications. Longan ECs were extracted using the epicatechin method.

The following chromatographic conditions were used. The assay was performed on a Diamonsil C18 (4. 6 mm × 200 mm, 5 μm) column with an isocratic elution in methanol: 0.1% acetic acid (34:66) at a flow rate of 1.0 mL/min. The column temperature was set at 30°C. All standards and samples were detected by UPLC (ACQUITY UPLC H-Class) at a wavelength of 257 nm.

### 2.6 Expression analysis by quantitative real-time PCR

Total RNAs were extracted from longan ECs grown under different light treatments using the TriPure Isolation Reagent (Roche Diagnostics, Indianapolis, IN, USA). The RNA quantities were determined using an Agilent 2100 bioanalyzer and were checked for denaturation by agarose gel electrophoresis. RNAs were stored at −80°C for later use. Total RNAs from longan ECs were reverse-transcribed and used for qPCR in accordance with previous methods [26,27]. Expression profiles of miRNAs were determined using an NCode Express SYBR GreenER miRNA qPCR kit (Invitrogen), and those of the mRNAs were determined using SYBR^®^ Premix Ex Taq^™^ II (Tli RNaseH Plus; Takara, Japan) on a LightCycler480 Real-time PCR system. *EF -1a*, *elF-4a* and *DlFSD1a* acted as the reference genes, and the relative gene expression levels were evaluated using the method described by Lin et al. [26]. dlo-miR156c, dlo-miR2089*-1 and 5S rRNA were used as the reference genes for miRNAs, and the relative miRNA expression levels were evaluated using the method described by Lin et al. [27]. Relative expression levels were determined using the 2^−ΔΔCt^ method. The quantitative PCR primers’ information is listed in S1 Table. Each treatment was analyzed using biological and technical triplicates.

### 2.7 Statistical analysis

Quantitative results of the gene expression and flavonoid contents analyses were presented in terms of means ± SDs of at least three biological replicates. The effect of different light conditions on the flavonoid contents and the gene expression were analysed by one-way analysis of variance (ANOVA) followed by Duncan’s test using SPSS version 19.0. These pictures were made using the GraphPad Prism 6.0 software.

## 3 Results

### 3.1 Influence of light quality, intensity, and photoperiod on the growth rate of longan ECs

**Fig 1:**
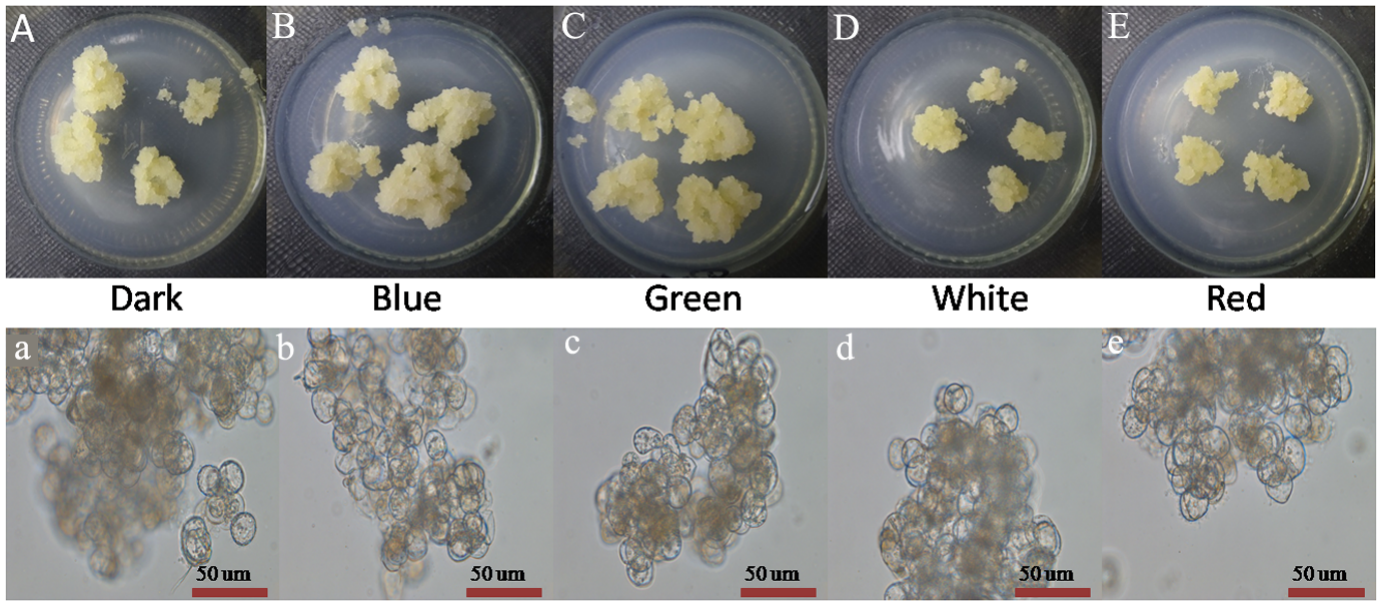
Growth state of longan ECs under different light qualities. A-E, Growth state of longan ECs on the 25 days under different light qualities, at the light intensity of 32 μmol ·m^-2^ ·s^-1^ and photoperiod of 12h; a-e, Growth situation of longan ECs on the 25 days under different light qualities by microscopic observation (Bar = 50nm).

The growth state of longan ECs was affected by light quality. Longan ECs in the dark, and under blue and green light conditions grew well, with a crunchy texture. But the growth status in the white and red light was poor. The texture mixed closely and the longan calli were watery with strong viscosity (Fig 1A–1E). Almost no difference was observed on the longan ECs under the different light qualities from cell microscopic observation (Fig 1a–1e).

**Fig 2:**
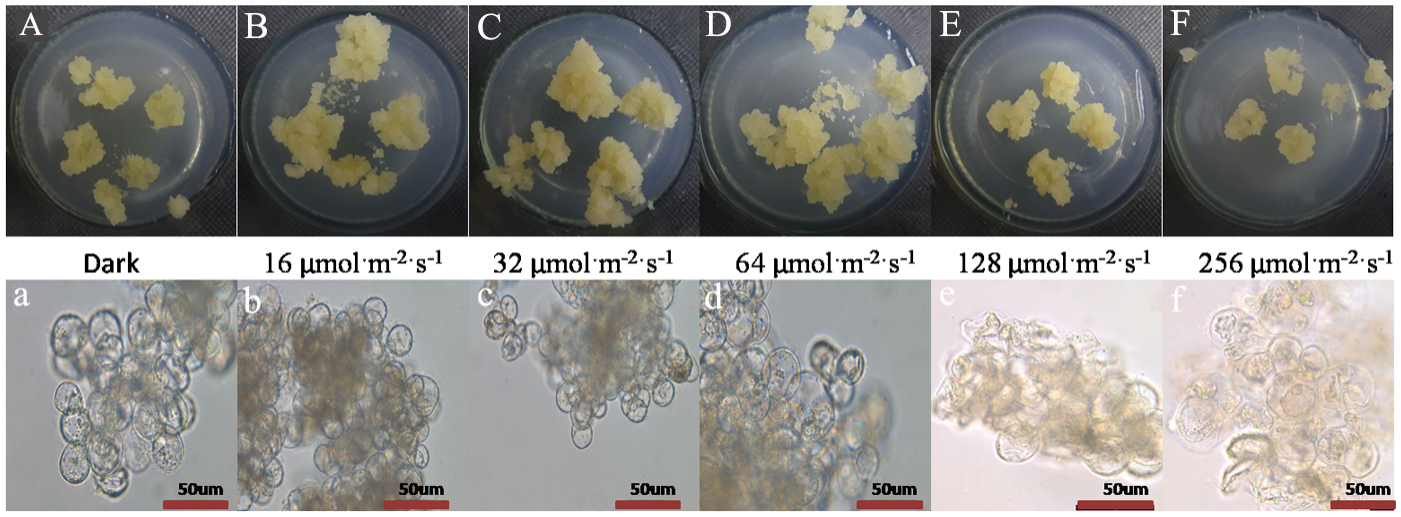
Growth state of longan ECs under blue light of different intensities. A-F, Growth state of longan ECs on the 25 days under blue light of different intensities, at photoperiod of 12h; a-f, Growth situation of longan ECs on the 25 days under blue light of different intensities by microscopic observation (Bar = 50nm).

The growth state of longan ECs was affected by different light intensities. The longan ECs under the dark, 16 μmol·m^-2^·s^-1^ to 64 μmol·m^-2^·s^-1^ were thrivinge, but the growth status under 128 μmol·m^-2^·s^-1^ to 256 μmol·m^-2^·s^-1^ was very poor and the structure was wet and sticky (Fig 2A–2F). The longan cytoplasm under the dark and 16 μmol·m^-2^·s^-1^ to 64 μmol·m^-2^·s^-1^ was all active. However, some cells under 128 μmol·m^-2^·s^-1^ to 256 μmol·m^-2^·s^-1^ showed cracking and deformation (Fig 2a–2f).

**Fig 3:**
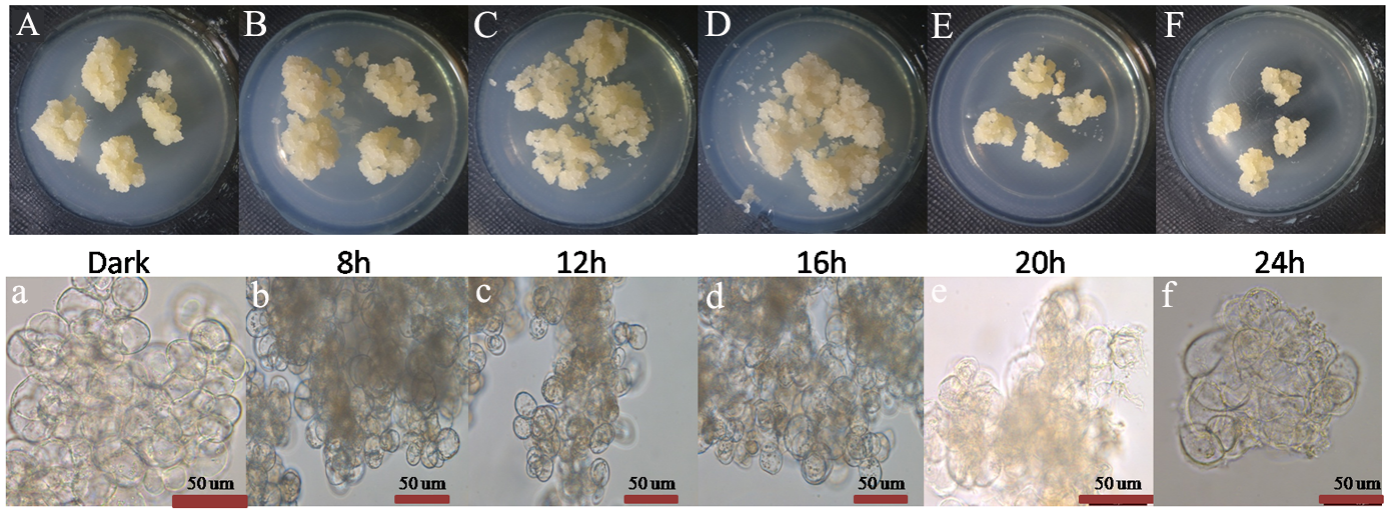
Growth state of longan ECs under blue light of different photoperiods. A-F, Growth state of longan ECs on the 25 days under blue light of different photoperiods, at light intensity of 32 μmol ·m^-2^ ·s^-1^; a-f, Growth situation of longan ECs on the 25 days under blue light of different photoperiods by microscopic observation (Bar = 50nm).

The growth state of longan ECs had significantly changed under different photoperiods. Compared to the growth in dark condition, longan ECs under dark, 8h, 12h, and 16h illumination were all thrive, but the growth state under 20h and 24h illumination were bad with waterish (Fig 3A–3F). Longan cytoplasm under dark and 8h to 16h illumination flow actively, but the cytoplasm under 20 h and 24 h illumination had poor fluidity, even some cracking and deformation were found in some cells (Fig 3a–3f).

**Fig 4:**
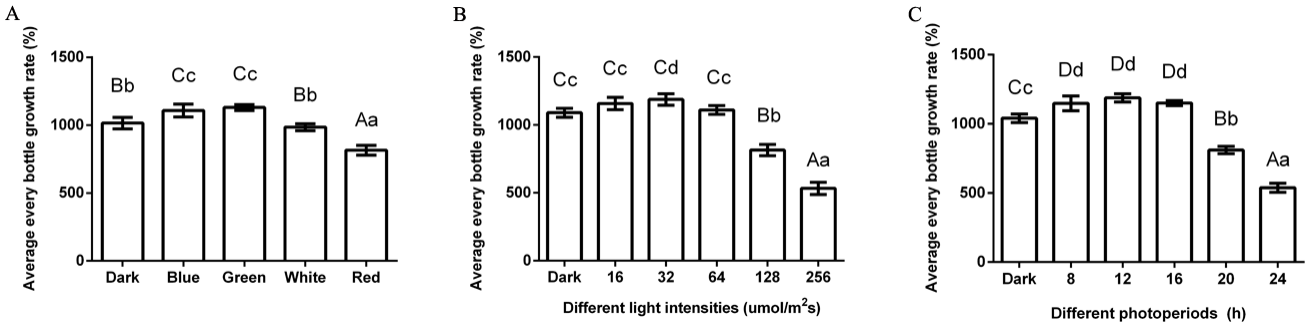
Growth rate of longan ECs under different lighting conditions. A, Growth rate of each bottle of Longan ECs on the 25 days under different light qualities; B, Growth rate of each bottle of Longan ECs on the 25 days under blue light of different intensities; C, Growth rate of each bottle of Longan ECs on the 25 days under blue light of different photoperiods; Values represent means ± SDs of three replicates. Different upper/lower case letters indicate statistically significant difference at 0.01/0.05 levels by one-way ANOVA analysis in the duncan’s test.

The growth rate of longan ECs was affected by light quality, intensity, and photoperiod. The growth rate of the longan ECs under blue and green light illumination was higher than that under the dark, but that under white and red light was even lower than that under the dark (Fig 4A and S2 Table). Longan ECs under 16 μmol·m^-2^·s^-1^ to 64 μmol·m^-2^·s^-1^ were higher than that under darkness. However, it was reduced significantly under 256 μmol·m^-2^·s^-1^ (Fig 4B and S3 Table). The grown rate of longan ECs under duration from 8 h to 16 h were higher than other treatment (Fig 4C and S4 Table). Generally, from the results above, longan ECs had a high growth rate in the condition of blue or green light, light intensity ranging from 16 μmol·m^-2^·s^-1^ to 64 μmol·m^-2^·s^-1^ and photoperiod ranging from 8 to 16 h.

### 3.2 Influence of light quality, intensity, and photoperiod on the flavonoid metabolism of longan ECs

**Fig 5:**
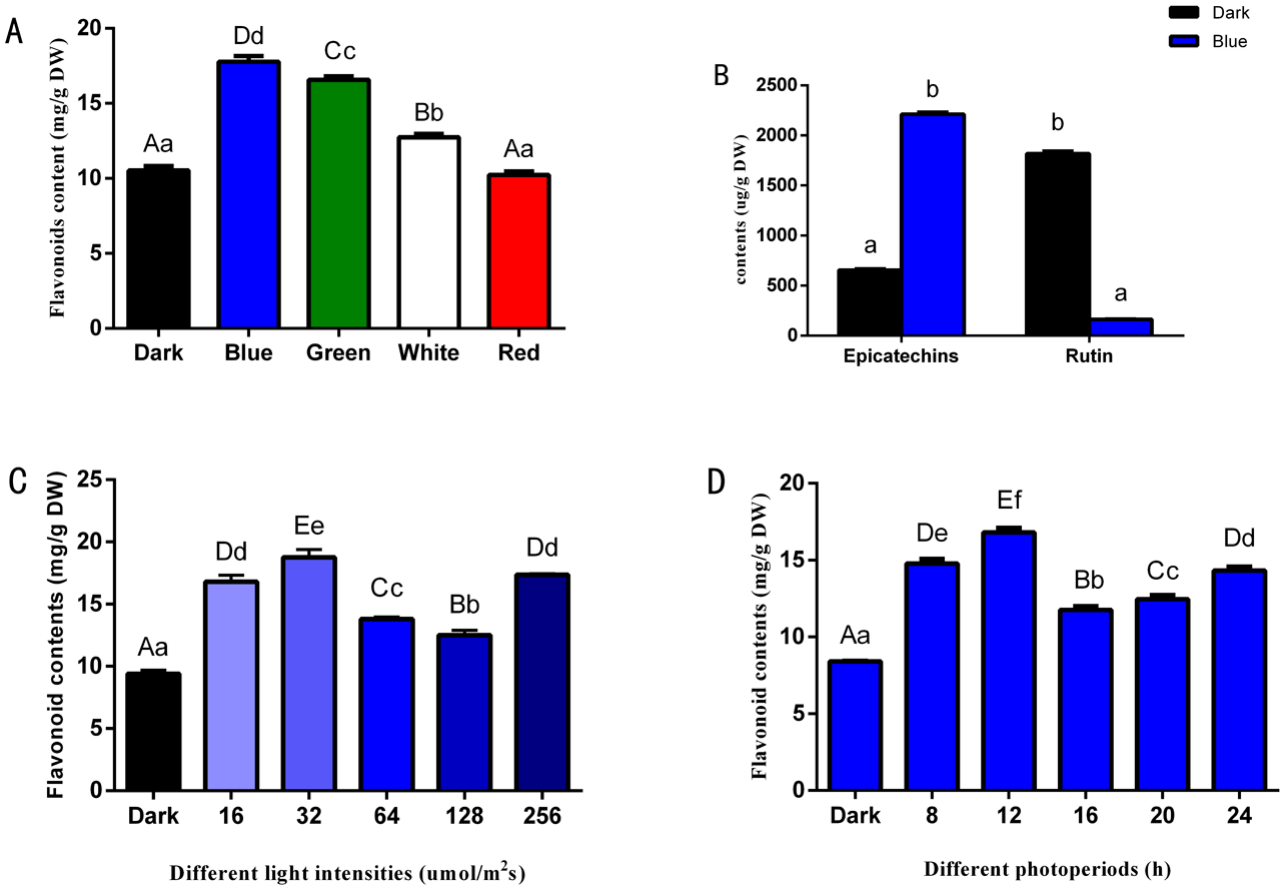
Flavonoid metabolites of longan ECs under different lighting conditions. A, Flavonoid contents in longan ECs under different light qualities, at the light intensity of 32 μmol ·m^-2^ ·s^-1^ and photoperiod of 12h; B, Flavonoid compound contents of longan ECs under different light qualities, light intensity of 32 μmol ·m^-2^ ·s^-1^, at photoperiod of 12h/d; C, Flavonoid contents in longan ECs under blue light of different intensities, at photoperiod of 12h/d; D, Flavonoid contents in longan ECs under blue light of different photoperiods, at light intensity of 32 μmol ·m^-2^ ·s^-1^; Values represent means ± SDs of three replicates. Different upper/lower case letters indicate statistically significant difference at 0.01/0.05 levels by one-way ANOVA analysis in the duncan’s test.

In our lab’s previous study, we compared flavonoid contents in longan ECs in different periods, and found that flavonoid contents peaked on 25^th^ day. [22]. Here, the flavonoid contents were determined in the longan ECs under different light qualities on 25^th^ day in this study. The highest content of flavonoids was 17.77 mg/g under blue light treatment, followed by the green light and a minimum value of 10.23 mg/g was measured under red light (Fig 5A and S5 Table). This indicated that blue light promoted the accumulation of epicatechin, but inhibited the synthesis of rutin (Fig 5B, S4 and S5 Figs, S6 and S7 Tables). Using the blue light of different intensities to cultivate longan ECs, flavonoid contents reached its highest at 18.77 mg/g when the light intensity was 32 μmol·m^-2^·s^-1^. Then the flavonoid contents was observed 12.51 mg/g, and 17.36 mg/g when the intensity was 256 μmol·m^-2^·s^-1^ (Fig 5C and S8 Table). When longan ECs were cultured under blue light at different photoperiods, the flavonoid contents was 16.81 mg/g in the photoperiod of 12 h/d. Then the flavonoid contents had a small rebound when the illumination lasted for 24 h (Fig 5D and S9 Table). In addition, flavonoid contents had a rebound under the light intensity of 256 μmol·m^-2^·s^-1^ (Fig 5C) and the illumination duration of 24 h/d (Fig 5D). These light conditions might have caused certain damage to longan ECs, which made they produce more flavonoids to respond to light stress by changing their physiological and metabolic mechanisms. In addition, the growth rate of red light treatment was lower compared with the control group (Fig 4A), whereas the flavonoid contents was not increased (Fig 5A). These reflected that red light conditions were not adverse to longan ECs, although red light was not suitable for longan ECs.

### 3.3 Flavonoid pathway genes expression level changes of longan ECs

**Fig 6:**
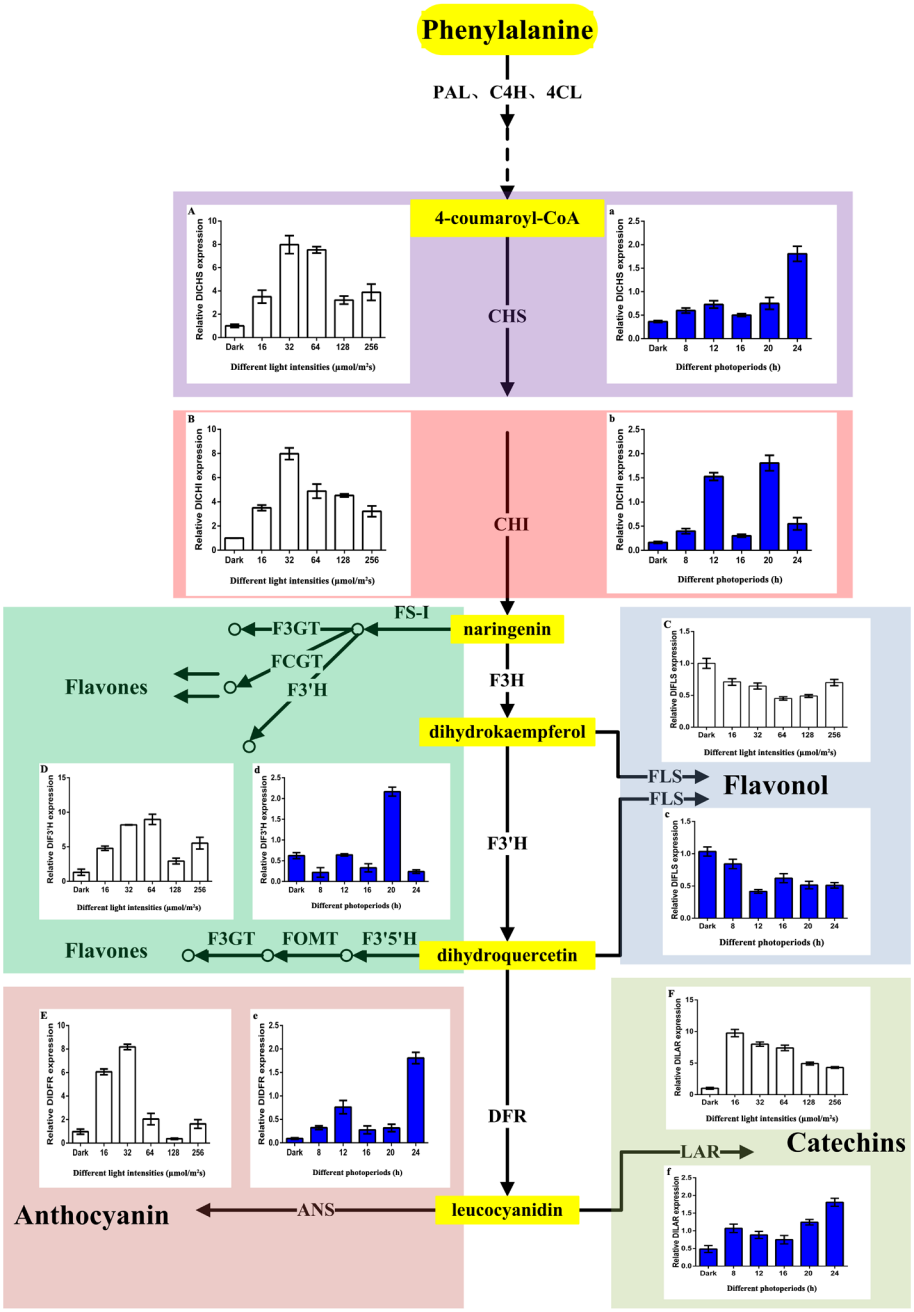
Flavonoid metabolic pathway genes of longan ECs under blue light. A-E, Flavonoid metabolic pathway genes of longan ECs under blue light of different intensities, at photoperiod of 12h/d; a-e, Flavonoid metabolic pathway genes of longan ECs under blue light of different photoperiods, at light intensity of 32 μmol ·m^-2^ ·s^-1^.

Real-time fluorescence quantitative analyses of the longan ECs flavonoid metabolic pathway genes under different blue light treatments were conducted. Six key genes were chosen from the flavonoid synthesis pathway: *CHS* and *CHI* are the first and second key genes, respectively, of the flavonoid synthesis pathway; *FLS* regulates flavonol compounds; *F3′H* has 35 family members in longan, which is the most members among the flavonoid pathway genes [14]; *DFR* is the key enzyme of anthocyanin synthesis; and *LAR* is the key gene of catechin synthesis [28] (Fig 6).

Flavonoids metabolic pathway genes of longan ECs were affected by blue light of different intensities, *DlCHS*, *DlCHI* and *DlDFR* reached their peak value under blue light of 32 μmol·m^-2^·s^-1^. However, the expression of *DlFLS* had maximum value under the dark treatment, and the expression of *DlFLS* was suppressed under blue light treatment (Fig 6 and S10 Table). Flavonoid metabolic pathway genes of longan ECs had significant changes under blue light of different photoperiods. *DlCHS*, *DlDFR* and *DlLAR* had the highest expression under photoperiod of 24h/d. This may be because longan EC was affected by adversity. The expression of *DlCHS*, *DlCHI* and *DlDFR* was the second under photoperiod of 12h/d. In addition, *DlFLS* had the highest expression under the dark treatment (Fig 6 and S11 Table). The results of flavonoid contents were confirmed through qPCR analysis by the key gene expression of flavonoid pathway (Figs 5 and 6).

### 3.4 Regulation of flavonoid metabolism in longan ECs by miR393, miR394 and miR395 under blue light conditions

To further understand the regulatory roles of miRNAs in light-affected flavonoid metabolism in longan ECs, their expression levels, including those of miR393, miR394 and miR395, and their target genes *DlTIR1-3*, *DlALMT12* and *DlAPS1*, respectively, as well as flavonoid pathway genes, were compared and analyzed. miR393, miR394 and miR395 are likely involved in the influence of light on longan ECs flavonoid metabolism. A psRNAtarget software-based analysis showed that miR393, miR394 and miR395 regulated *DlTIR1-3*, *DlALMT12* and *DlAPS1*, respectively. In addition, *CHS*, *CHI*, *FLS*, *F3′H*, *DFR* and *LAR* were the key genes of flavonoid pathway.

**Fig 7:**
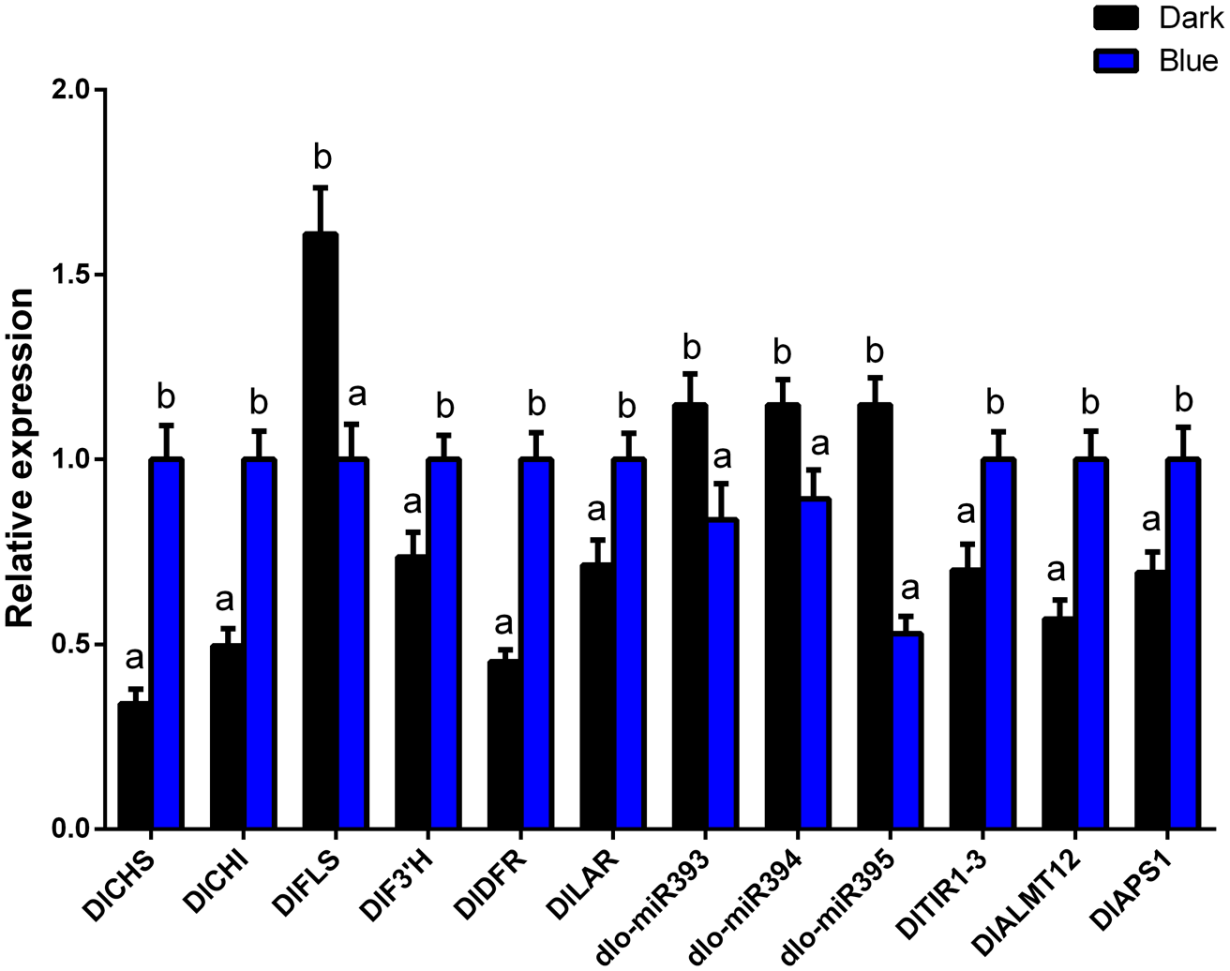
miRNAs and flavonoid metabolish related genes of longan ECs under blue light. Both light intensity and photoperiod were fixed at 32 μmol·m^-2^·s^-1^ and 12h/d respectively; Values represent means ± SDs of three replicates. Different lower case letters indicate statistically significant difference at 0.05 levels by one-way ANOVA analysis in the duncan’s test.

These key genes of flavonoid metabolism by qPCR analysis, compared with the dark group, flavonoid related key genes *DlCHS*, *DlCHI*, *DlF3′H*, *DlDFR*, *DlLAR* and target genes *DlTIR1-3*, *DlALMT12*, *DlAPS1* all showed up-regulated trend under blue light treatment. However, miR393, miR394, miR395 and flavonoid key genes *DlFLS* all showed down-regulated trend (Fig 7 and S12 Table). Here, we speculated that miR393, miR394, and miR395 acted on target genes, which negatively regulated flavonoid key genes *DlFLS* and positively regulated key genes *DlCHS*, *DlCHI*, *DlF3′H*, *DlDFR*, *DlLAR* under blue light (Fig 7 and S12 Table). The expression of anthocyanin and catechin synthetic related genes were increased, resulting in the decreased conversion of dihydrokaempferol to kaempferol by *FLS* and the content of flavonols (Figs 6 and 7). Therefore, when blue light was the most appropriate, it might promote the accumulation of anthocyanin, catechins, inhibite the synthesis of flavonols.

### 3.5 Analysis of miRNAs and target genes expression patterns under blue light of different intensities

**Fig 8:**
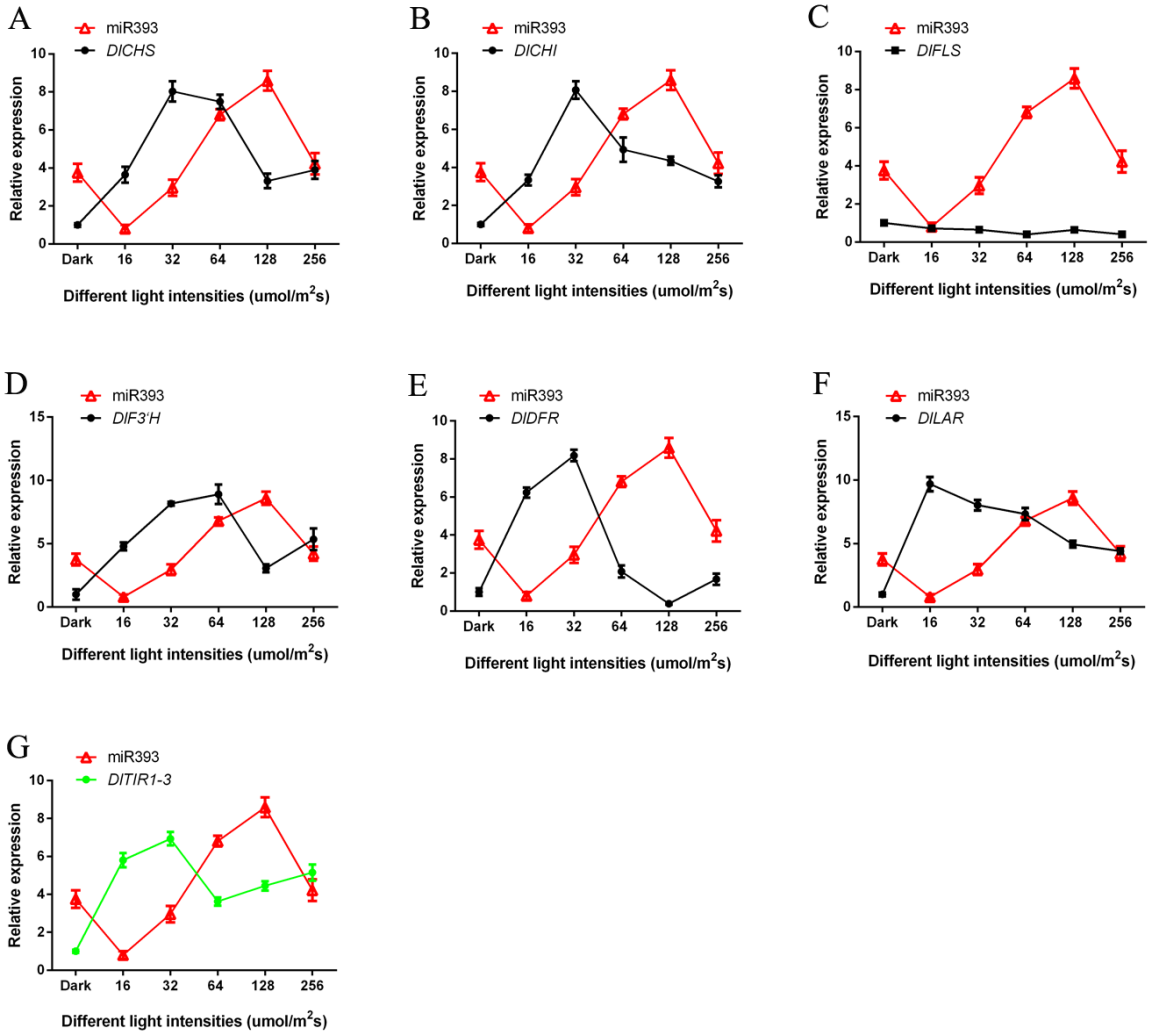
The relationship of miR393 and flavonoid metabolish related genes under blue light of different intensities. A, miR393 and *DlCHS*; B, miR393 and *DlCHI*; C, miR393 and *DlFLS*; D, miR393 and *DlF3′H*; E, miR393 and *DlDFR*; F, miR393 and *DlLAR*; G, miR393 and its target gene *DlTIR1-3*.

**Fig 9:**
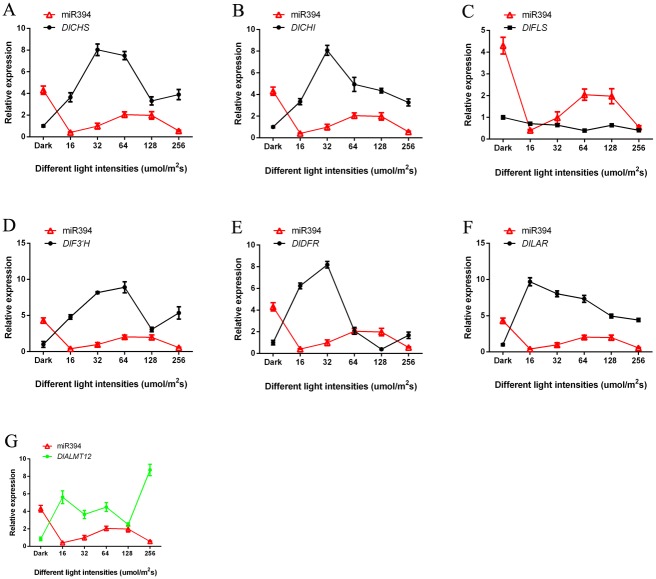
The relationship of miR394 and flavonoid metabolish related genes under blue light of different intensities. A, miR394 and *DlCHS*; B, miR394 and *DlCHI*; C, miR394 and *DlFLS*; D, miR394 and *DlF3′H*; E, miR394 and *DlDFR*; F, miR394 and *DlLAR*; G, miR394 and its target gene *DlALMT12*.

**Fig 10:**
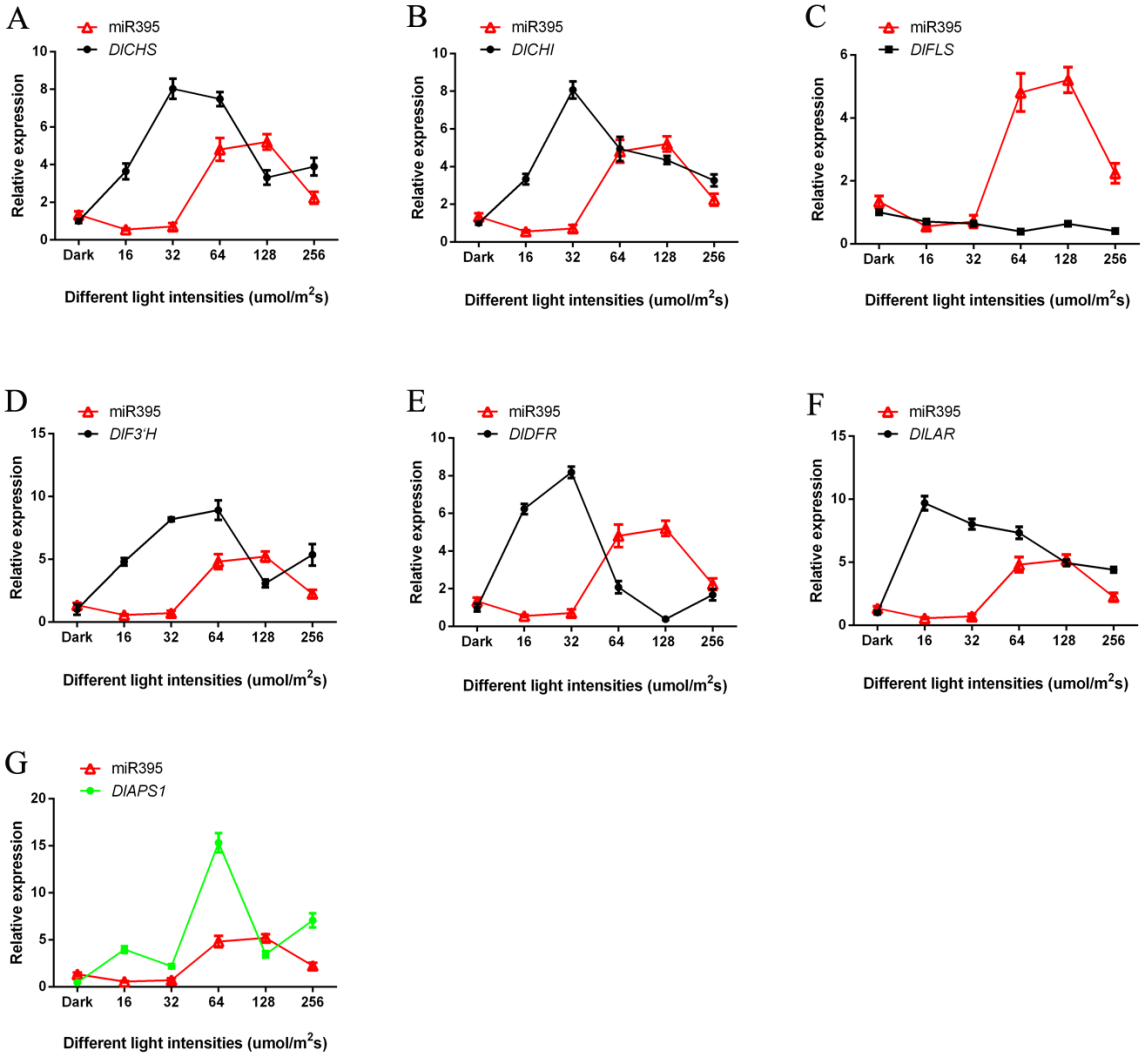
The relationship of miR395 and flavonoid metabolish related genes under blue light of different intensities. A, miR395 and *DlCHS*; B, miR395 and *DlCHI*; C, miR395 and *DlFLS*; D, miR395 and *DlF3′H*; E, miR395 and *DlDFR*; F, miR395 and *DlLAR*; G, miR395 and its target gene *DlAPS1*.

Flavonoid key genes of longan ECs under blue light of different intensities were analysised by qPCR. With the increase of the light intensity, the expression trend of miR393, miR394 and miR395 was basically the same (Figs 8–10 and S13 Table). The expression of miR393 under light intensity of 16 and 32 μmol·m^-2^·s^-1^ was lower than that under the dark. The expression of another intensity treatment was higher than dark treatment (Fig 8). However, the expression of miR394, miR395 under dark treatment was higher than that under blue light (Figs 9 and 10). This may be caused by the fact that different miRNAs had different expression patterns in response to blue light of different intensities.

In this study, the trend of their expressions (miR393 and target gene *DlTIR1-3*, miR394 and target gene *DlAlMT12*, miR395 and target gene *DlAPS1*) presented negative correlation under blue light of different intensities in longan ECs (Figs 8–10 and S13 Table). This also confirmed that miR393, miR394, miR395 and target genes had a negative regulating relationship under blue light of different intensities in longan ECs. However, individual stages of the expression of miRNAs and target genes showed the same trend under blue light of different intensities. For example, miR393 target *DlTIR1-3* during 16 μmol·m^-2^·s^-1^ to 32 μmol·m^-2^·s^-1^ and 64 μmol·m^-2^·s^-1^ to 128 μmol·m^-2^·s^-1^ (Fig 8). This might be the reason that a target gene was regulated by different members of the miRNAs family or other miRNAs in plant response to different light conditions. These miRNAs had their own role under blue light at different intensities. While some miRNA members could not regulate the expression of mRNA, other members complemented their functions and finally regulate the target genes.

The miR393, miR394, miR395 and *DlFLS* basically presented positive correlation, but the other flavonoid pathway gene *DlCHS*, *DlCHI*, *DlF3′H*, *DlDFR* and *DlLAR* basically presented a negative correlation under the blue light of different intensities treatment (Figs 8–10 and S13 Table). This indicated that miRNAs had different regulation methods for different genes in the pathway of flavonoids. So, as was shown in Fig 2B, the miR393, miR394, and miR395 promoted the synthesis of epicatechin, but inhibited the synthesis of rutin.

### 3.6 Analysis of miRNAs and target genes expression patterns under blue light of different photoperiods

**Fig 11:**
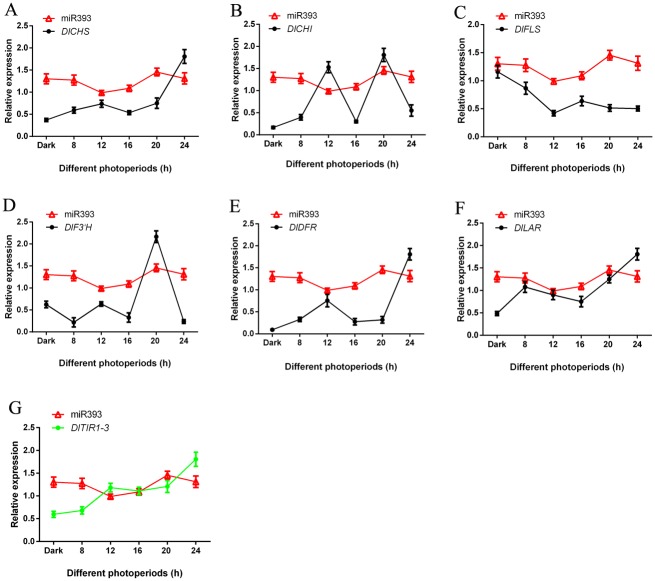
The relationship of miR393 and flavonoid metabolish related genes under blue light of different photoperiods. A, miR393 and *DlCHS*; B, miR393 and *DlCHI*; C, miR393 and *DlFLS*; D, miR393 and *DlF3′H*; E, miR393 and *DlDFR*; F, miR393 and *DlLAR*; G, miR393 and its target gene *DlTIR1-3*.

**Fig 12:**
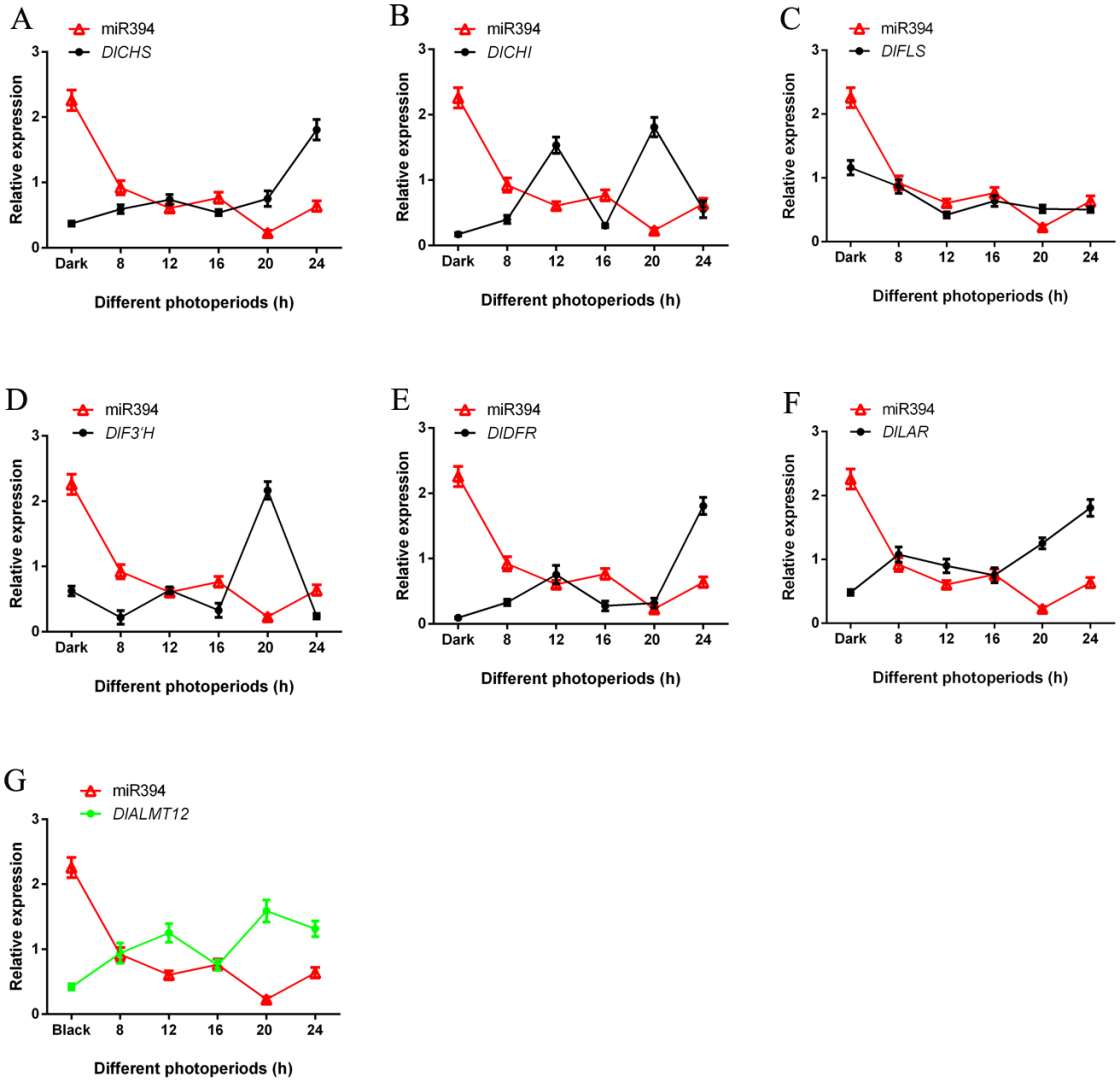
The relationship of miR394 and flavonoid metabolish related genes under blue light of different photoperiods. A, miR394 and *DlCHS*; B, miR394 and *DlCHI*; C, miR394 and *DlFLS*; D, miR394 and *DlF3′H*; E, miR394 and *DlDFR*; F, miR394 and *DlLAR*; G, miR394 and its target gene *DlALMT12*.

**Fig 13:**
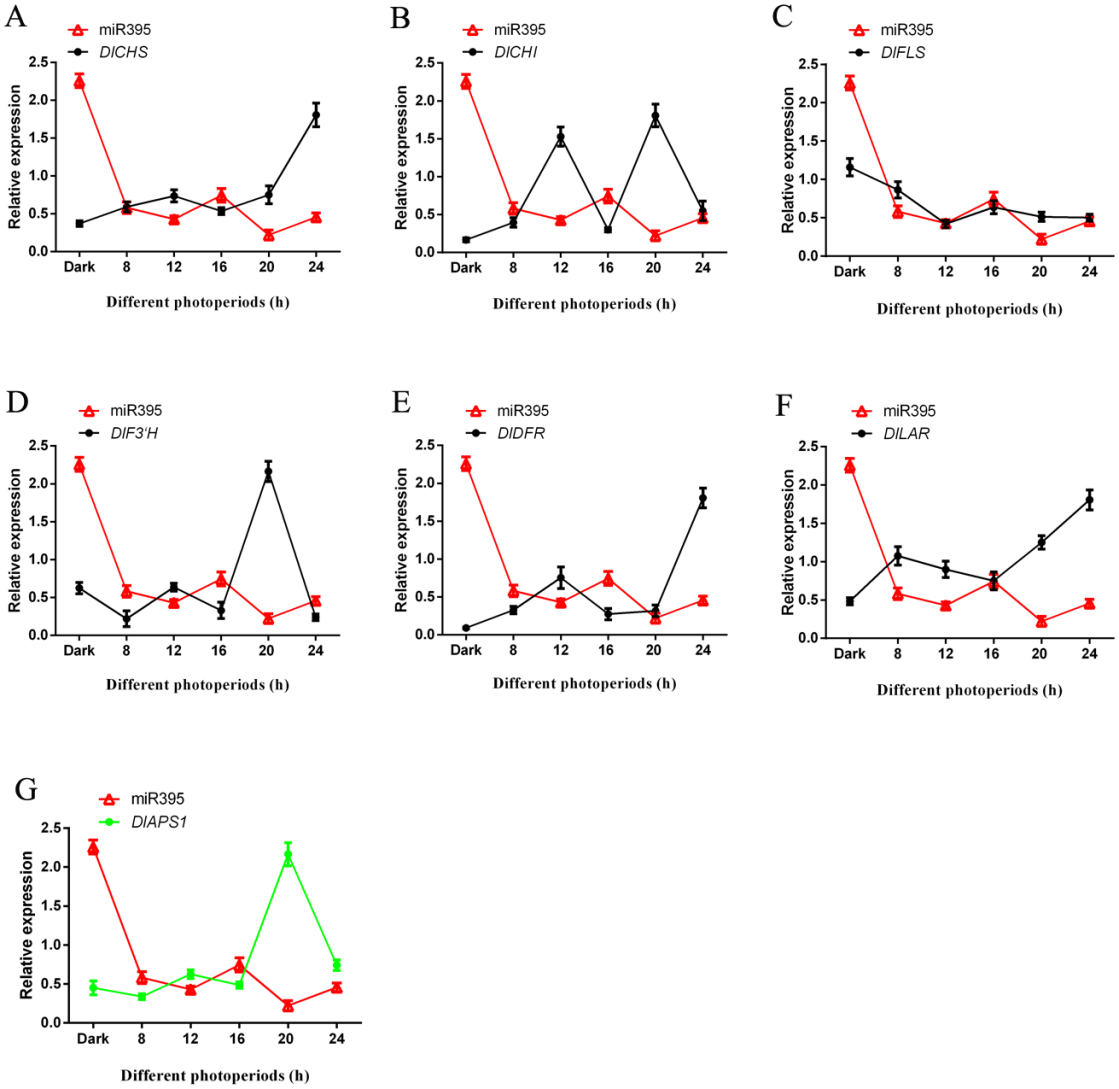
The relationship of miR395 and flavonoid metabolish related genes under blue light of different photoperiods. A, miR395 and *DlCHS*; B, miR395 and *DlCHI*; C, miR395 and *DlFLS*; D, miR395 and *DlF3′H*; E, miR395 and *DlDFR*; F, miR395 and *DlLAR*; G, miR395 and its target gene *DlAPS1*.

Flavonoid key genes of longan ECs under blue light of different photoperiods were analyzed by qPCR. With the extension of photoperiod, the expression trend of miR393, miR394 and miR395 was basically the same (Figs 11–13 and S14 Table). The expression of miR393 had small difference in the dark and blue light treatment (Fig 11). However, the expression of miR394, miR395 under dark treatment was much higher than that under blue light of different photoperiods (Figs 12 and 13).

In this study, the trend of their expression (miR393 and target gene *DlTIR1-3*, miR394 and target gene *DlAlMT12*, miR395 and target gene *DlAPS1*) presented negative correlation under blue light of different photoperiods in longan ECs (Figs 11–13 and S14 Table). This also confirmed that miR393, miR394 and miR395 and target genes had a negative regulating relationship under blue light of different photoperiods in longan ECs. In addition, individual stages of the expression of miR393, miR394, miR395 and target genes also showed the same trend under blue light of different photoperiods.

In this treatment, the expression of miR393, miR394, miR395 and flavonoid key genes *DlFLS* had basically the same trend. However, the other flavonoid pathway gene *DlCHS*, *DlCHI*, *DlF3’ H*, *DlDFR* and *DlLAR* basically presented an opposite trend (Figs 11–13 and S14 Table). The expression trends of the longan flavonoids pathway gene and miR393, miR394, and miR395 were not exactly the same. This may be the reason that the longan flavonoid pathway gene contained multiple family members. For example, *DlCHS* had 14 family members and *DlDFR* had 4 family members and so on [14]. In this study, while some family members were unable to express at one stage of blue light, other family members might complement each other.

**Fig 14:**
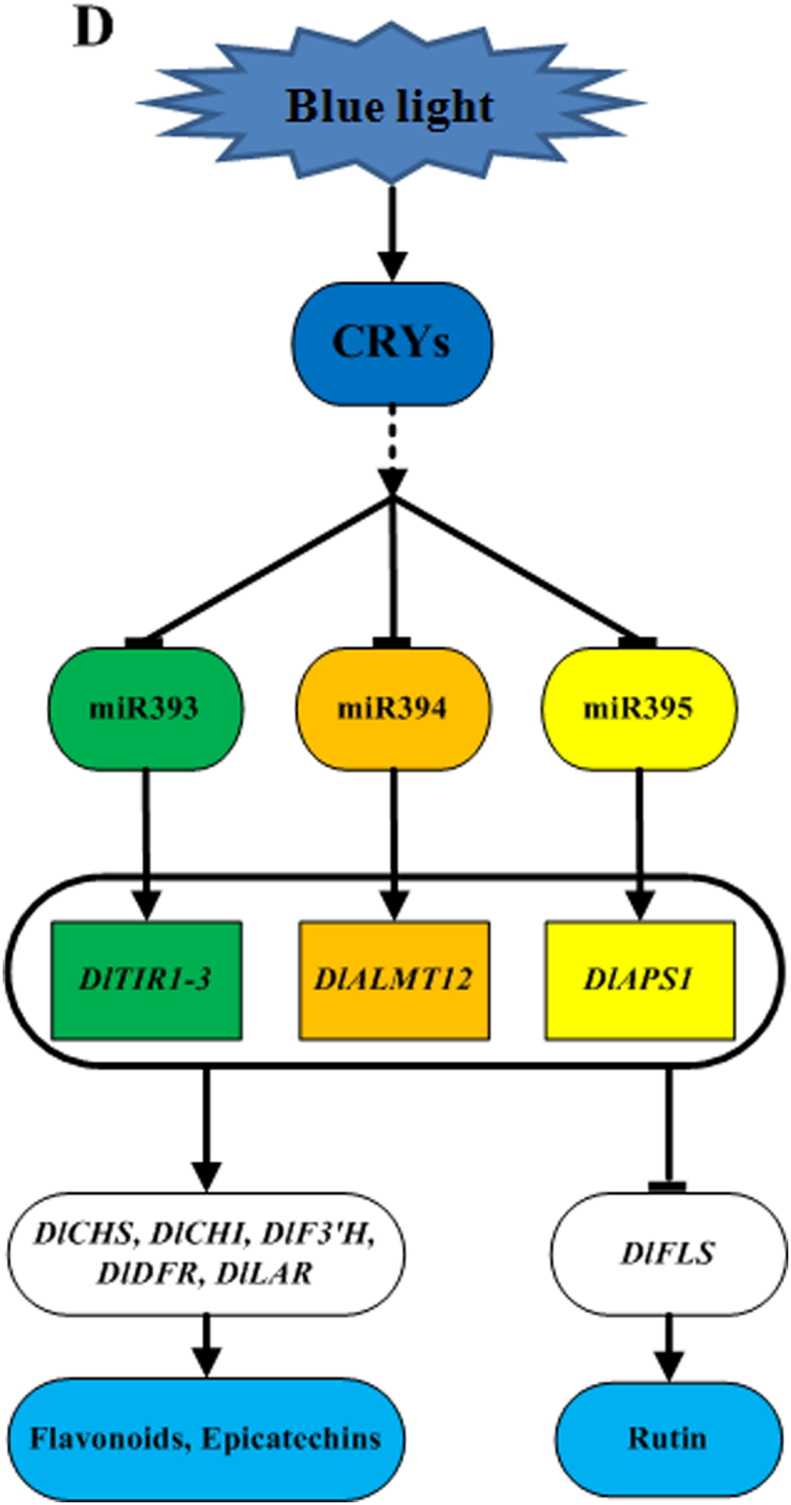
The relationship of miR393, miR394, miR395 and flavonoid metabolism under blue light. Flavonoid metabolic pathway genes: *DlCHS*, *DlCHI*, *DlFLS*, *DlF3′H*, *DlDFR*, *DlLAR*; Target genes: *DlTIR1-3*, *DlALMT12*, *DlAPS1*.

In conclusion, these results indicated that miR393 and its target gene *DlTIR1-3*, miR394 and its target gene *DlAlMT12*, and miR395 and its target gene *DlAPS1* had a negative regulating relationship under blue light in longan ECs. Furthermore, miR393, miR394, and miR395 acted on target genes, which negatively regulated flavonoid key genes *DlFLS* and positively regulated key genes *DlCHS*, *DlCHI*, *DlF3′H*, *DlDFR*, *DlLAR*, and finally affected the accumulation of flavonoids. The treatment of longan ECs under the blue light at the intensity of 32 μmol·m^-2^·s^-1^ for 12 h/d inhibited the expression of miR393, miR394 and miR395, which promoted the expression of target genes and the accumulation of flavonoids and epicatechin, but inhibited the synthesis of rutin (Fig 14). This kind of regulation mechanism may need a further verification using genetic transformations.

## 4 Discussion

### 4.1 Blue light was conducive to flavonoid accumulation in longan ECs

Controlling the quality of light is an effective means of secondary metabolites synthesis [18]. Changes in flavonoid contents are related to different light qualities and can be promoted by blue light or inhibited by red light in cell cultures of *Saussurea medusa* ‘Maxim’ [29]. Other studies on *Saccharina japonica* [30] and lettuce [31] also indicate that blue light promotes flavonoid synthesis. Thus, shorter wavelength conditions are more conducive to the accumulation of flavonoids [7]. Here, the longan EC flavonoid contents’ trend was blue light > green light > dark > white light > red light. The results also indicated that flavonoid synthesis was related to blue-light induction and connected to the absorption spectra of flavonoid substances in longan ECs. Moreover, metabolic-related gene expression levels were significantly higher than under other light-quality treatments. This supports the conclusion that short wavelength light is more efficient for flavonoid synthesis.

Different light qualities can be effectively used on plants under the appropriate optical density, termed photosynthetic photon flux density, conditions [32]. Thus, the secondary metabolism of plant cells can be controlled by the photosynthetic photon flux density. In the determination of flavonoid contents under different intensities of blue light, 32 μmol·m^−2^·s^−1^ was the most beneficial to the growth of, and flavonoid accumulation in, longan ECs. These results were in accordance with the flavonoid key gene expression results. Based on the different plant growth needs and characteristics, an appropriate light intensity can be selected for culturing. The carotenoid contents are higher under medium light intensity (270 μmol·m^−2^·s^−1^) followed by low (175 μmol·m^−2^·s^−1^) and high (450 μmol·m^−2^·s^−1^) in orchid tissue-cultured seedlings [33]. In tropical mangosteen (*Garcinia mangostana*) fruit [34], anthocyanin accumulation is not stimulated by light, and light can even decrease anthocyanin biosynthesis in pears [35]. Thus, the light intensity range is important, especially in plant cultivation, and even affects the repeatability and accuracy of experiments. In general, longan ECs are cultured in the dark. Therefore, the weak light intensity of 32 μmol·m^−2^·s^−1^ was adopted to study light effects on longan ECs.

The circadian rhythms of plant cells are affected by photoperiod, which effects the synthesis of secondary metabolites [36]. For the determination of flavonoid contents in longan ECs under different photoperiods of blue light, a photoperiod of 12 h/d was found most effective for the growth of, and flavonoid accumulation in, longan ECs. Flavonoid contents and key gene expression levels showed increasing trends with a photoperiod of 24 h/d. However, a significant reduction in the longan EC’s growth was observed owing to light stress. Almost all metabolic pathways include at least one enzyme that is under circadian transcriptional control [37]. Therefore, appropriately shortened or extended photoperiod for plants can change the contents of their secondary metabolites [38].

### 4.2 Regulatory miRNA-mediated mechanisms of flavonoid biosynthesis under blue light

Genes related to light-inducible flavonoid metabolic pathways have been widely reported [7]. However, investigations into the roles of miRNAs in light-mediated flavonoid metabolism are still lacking. Recently, ‘Omics’ analyses of the relevant miRNAs revealed that light affected secondary metabolic pathways. Integrated RNA seq and sRNA seq analyses have indicated that the regulation of miRNAs is closely linked to light-affected secondary metabolites in potato [39]. In agarwood, genes related to secondary metabolism were regulated under red light/far-red conditions, which are associated with the regulation of miRNAs and DNA methylation [40]. The results of this study were also confirmed that miR393, miR394 and miR395 acted on target genes and affected the accumulation of flavonoids under blue light condition in longan ECs.

In previous studies, researchers found that both miR393 [16] and *TIR1*[41] could regulate plant’s secondary metabolism. *TIR1* was a key factor in the plant auxin signal transduction pathway[42]. The synthesis rate of flavonoids was improved by auxin, and there was a feedback effect between flavonoids and auxin [43]. Meanwhile, *DlTIR1* promoter contained a lot of light reaction-related components [44] and flavonoid metabolism was regulated by *DlTIR1* in light response. In addition, the fact of miR393 regulated target gene *DlTIR1-3* and the model of the negative correlation between their expressions were confirmed by Lai et al [43]. In conclusion, miR393 acted on *DlTIR1-3*, then affected the accumulation of flavonoids under blue light conditions.

In our studies, the fact of miR394 regulated target gene *DlALMT12* was also proved through psRNAtarget software analysis. As the blue light changes, miR394 and *DlALMT12* present a negative correlation. In addition, *ALMT* have important regulation function in the synthesis of malic acid in the TCA [45]. In higher plants, secondary metabolism is intermediate product derived from primary metabolism tricarboxylic acid cycle (TCA), glycolysis system (EMP), pentose phosphate pathway (PPP) et al [46]. In short, miR394 acted on *DlALMT12* and then further influenced the accumulation of flavonoids under blue light.

It was reported that miR395 could shear *APS1* specifically. The over-expression of miR395 results in the *APS1* unable to receive external signal stimulation [47]. The miR395 regulated target gene *DlAPS1* was also proved through psRNAtarget software analysis in longan EC. As the blue light changes, miR395 and *DlAPS1* present a negative regulatory correlation. Sulfate assimilation is a key component in both primary and secondary metabolisms. An essential step in this pathway is the activation of sulfate through adenylation by the enzyme ATP sulfurylase (ATPS), forming adenosine 5’-phosphosulfate (APS) [48]. In brief, miR395 targeted *DlAPS1*, then influenced flavonoid synthesis under blue light conditions.

The miRNAs can act on the genes encoding its rate-limiting enzyme, and also act on transcription factors, then regulate the secondary metabolism of plants [49]. Degradome sequencing of the tea (Camellia sinensis) miRNA target genes indicates that Csn-miR167a targets *CHI* and Csn-miR2593e targets *ANR* then regulates the accumulation of flavonoids [50]. However, miR393, miR394, and miR395 acted on transcription factors and regulated flavonoid synthesis in this study. Transcription factors can be used as target genes of miRNAs, and the function of some transcription factors involves the transduction of plant hormone signals. Secondary metabolism is closely related to hormone signal transduction [51]. *Arabidopsis* miR393 can act on auxin response factors 1 and 9, and thus, regulates glucosinolate synthesis [19]. The negative regulation of anthocyanin biosynthesis by a miR156-targeted *SPL* transcription factor (*SPL* involves the transduction of hormone signals) was described by Gou et al [20]. Another type of transcription factor may directly participate in the blue light signal network, thus affecting the synthesis of flavonoids. The blue light signal network has been drawn in the study of *Arabidopsis* [52]. Therefore, it is further verified that the target gene of miRNAs may participate in the blue light signal network and thus regulates the metabolism of flavonoids.

The miRNAs may affect the expression levels of multiple structural genes in the flavonoid pathways, and the effects of miRNAs may be more obvious than those of certain flavonoid-related genes [15]. miRNAs are important regulatory factors in plants and help mediate the influence of light signals on flavonoid metabolism.

Thus, miR393, miR394, and miR395 played an important role in the flavonoid metabolism of longan ECs under blue light conditions, which needed to be further verification using genetic transformations.

## 5. Conclusion

In our study, miR393 and its target gene *DlTIR1-3*, miR394 and its target gene *DlAlMT12*, and miR395 and its target gene *DlAPS1* had a negative regulating relationship under blue light in longan ECs. Furthermore, miR393, miR394, and miR395 acted on target genes, which negatively regulated flavonoid key genes *DlFLS* and positively regulated key genes *DlCHS*, *DlCHI*, *DlF3′H*, *DlDFR*, *DlLAR*, and finally affected the accumulation of flavonoids. The miRNAs played an important role in the flavonoids metabolism of longan ECs regulated by blue light, which showed that light regulated the molecular mechanism of flavonoids metabolism in plants.

## Supporting information

S1 Fig Plant growth chamber. (TIF)

S2 Fig LED light. (TIF)

S3 Fig Illumination uniformity analysis of LED light. (TIF)

S4 Fig Epicatechin contents of longan ECs under different treatments. A, dark treatment; B, blue light treatment; 1, epicatechin.
(TIF)

S5 Fig Rutin contents of longan ECs under different treatments. A, dark treatment; B, blue light treatment; 2, rutin.
(TIF)

S1 Table Primers information used for real-time PCR analysis of longan genes. (DOCX)

S2 Table Growth rate of each bottle of longan ECs on the 25 days under different light qualities. (DOCX)

S3 Table Growth rate of each bottle of longan ECs on the 25 days under blue light of different intensities. (DOCX)

S4 Table Growth rate of each bottle of longan ECs on the 25 days under blue light of different photoperiods. (DOCX)

S5 Table Flavonoid contents of longan ECs under blue light of different quality. (DOCX)

S6 Table Epicatechin contents of longan ECs under different treatments. (DOCX)

S7 Table Rutin contents of longan ECs under different treatments. (DOCX)

S8 Table Flavonoid contents of longan ECs under blue light of different intensities. (DOCX)

S9 Table Flavonoid contents of longan ECs under blue light of different photoperiods. (DOCX)

S10 Table The expression of flavonoid metabolic pathway genes of longan ECs under blue light of different intensities. (DOCX)

S11 Table The expression of flavonoid metabolic pathway genes of longan ECs under blue light of different photoperiods. (DOCX)

S12 Table The expression of miRNAs and flavonoid metabolish related genes of longan ECs under blue light. (DOCX)

S13 Table The expression of miRNAs and flavonoid metabolish related genes under blue light of different intensities. (DOCX)

S14 Table The expression of miRNAs and flavonoid metabolish related genes under blue light of different photoperiods. (DOCX)
