# Supplementary figures and images for: Effects of blue light on flavonoid accumulation linked to the expression of miR393, miR394 and miR395 in longan embryogenic calli
Source: PLoS One. 2018 Jan 30;13(1):e0191444. doi: 10.1371/journal.pone.0191444 (PMC5790225; doi:10.1371/journal.pone.0191444)

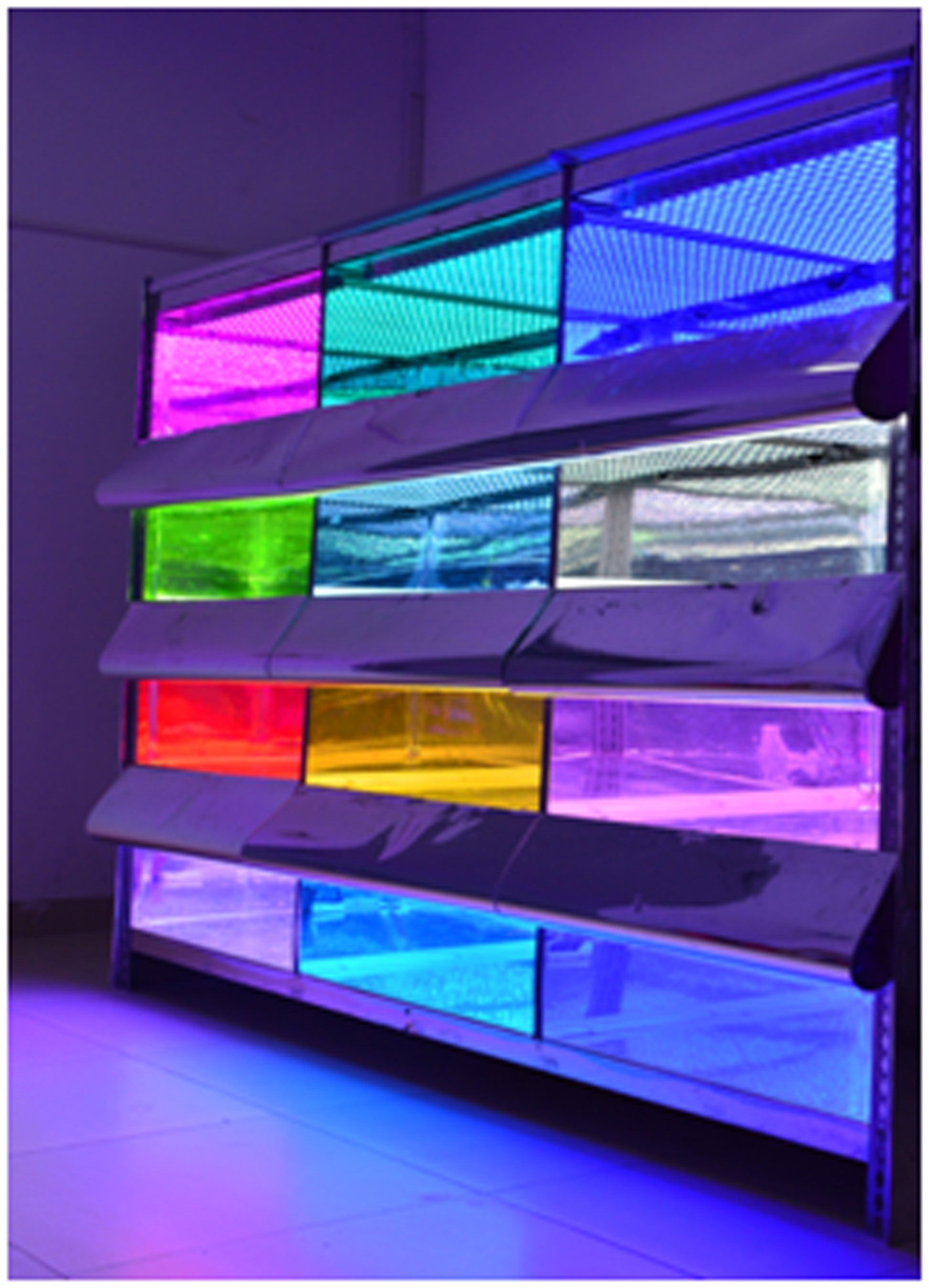

Supplement: S1 Fig — (TIF) [file pone.0191444.s001.tif]

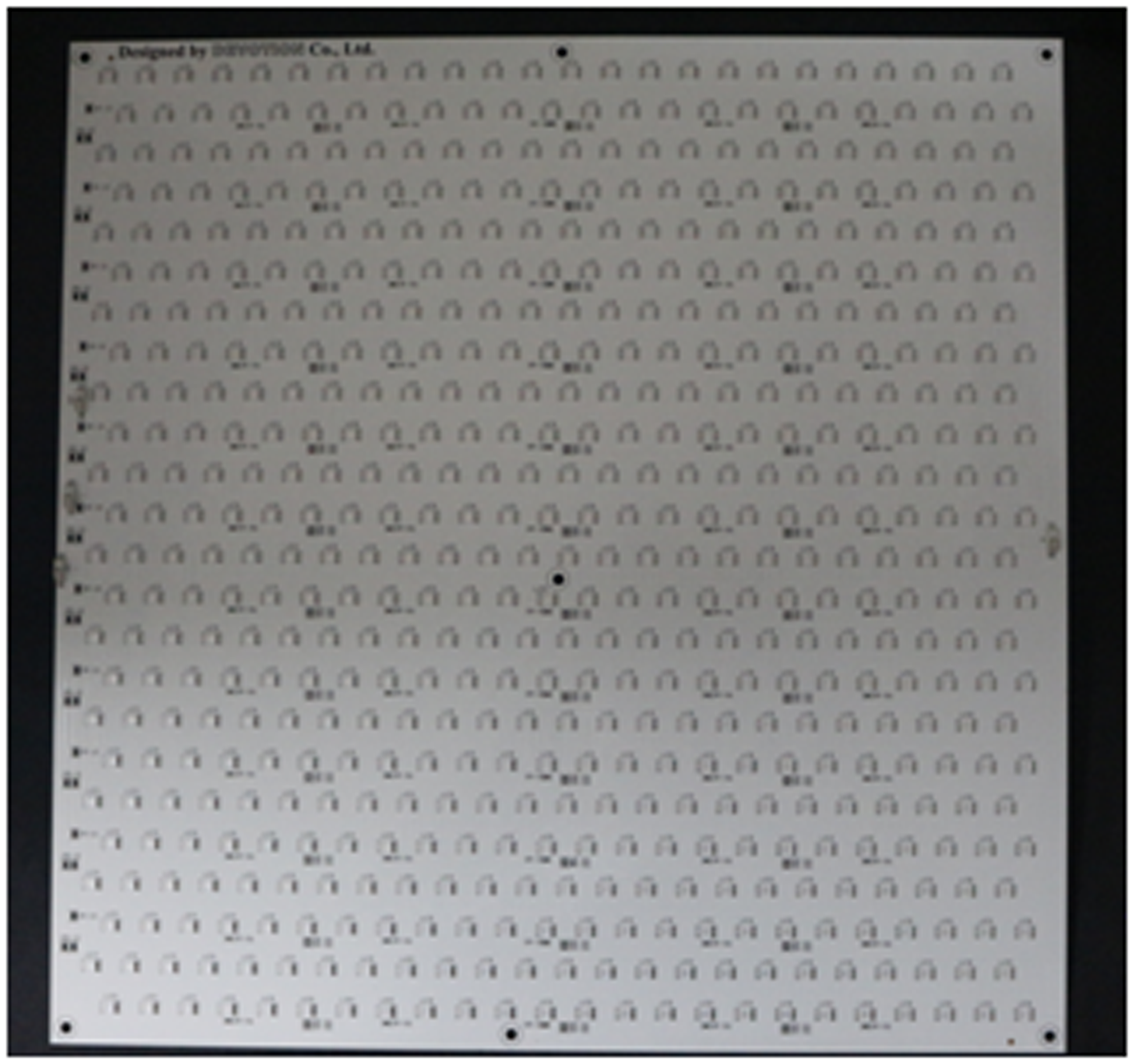

Supplement: S2 Fig — (TIF) [file pone.0191444.s002.tif]

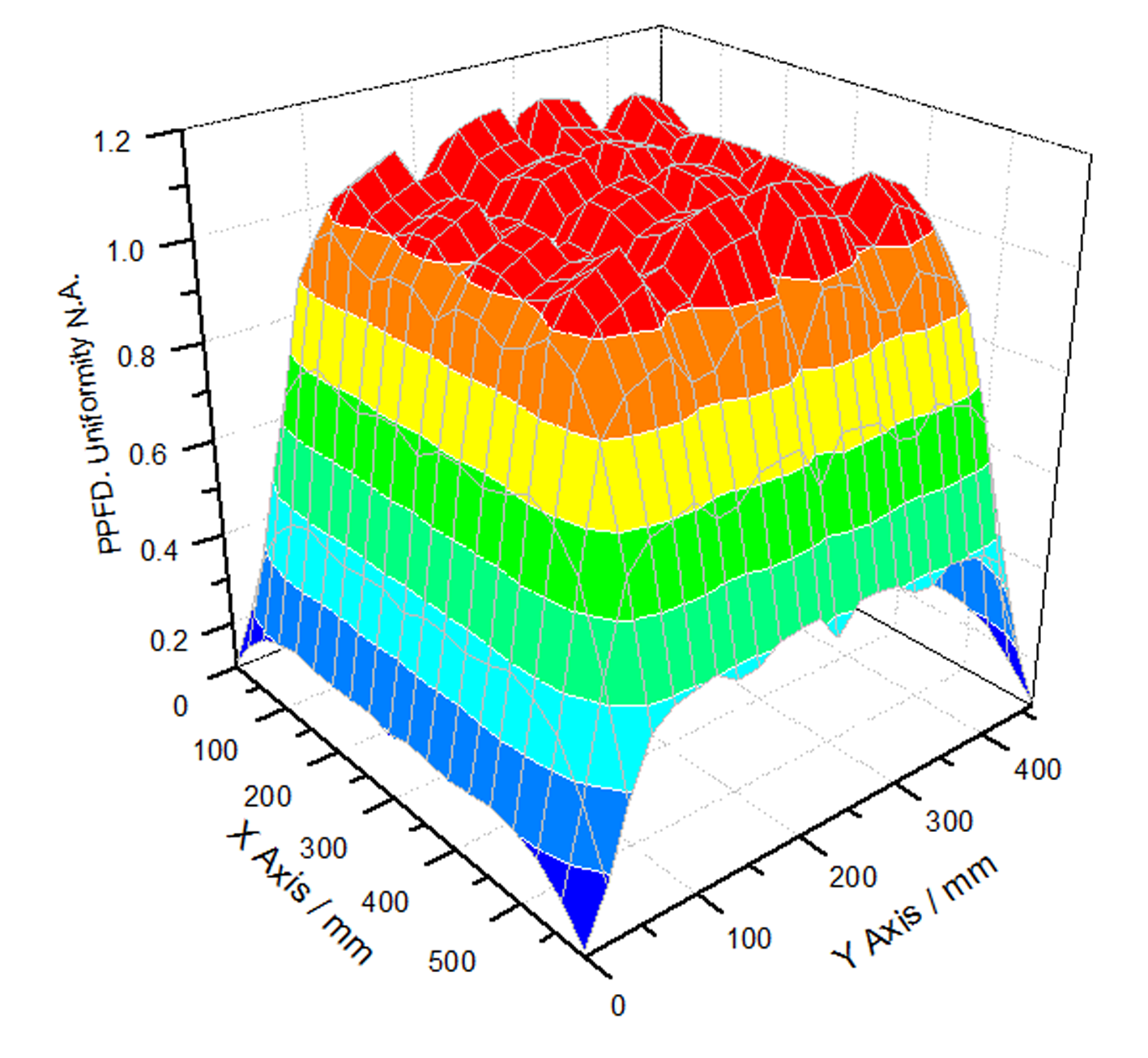

Supplement: S3 Fig — (TIF) [file pone.0191444.s003.tif]

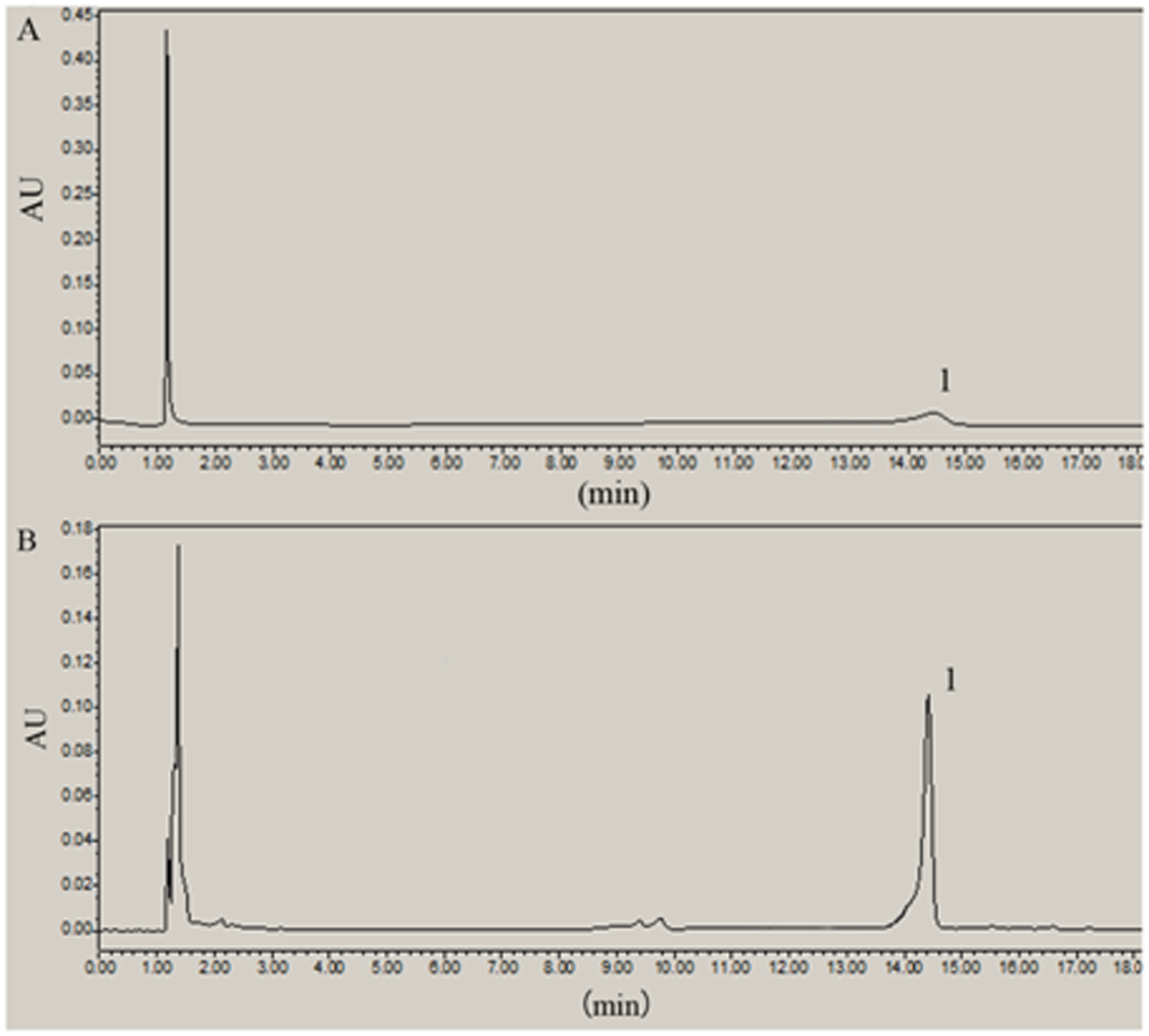

Supplement: S4 Fig — A, dark treatment; B, blue light treatment; 1, epicatechin. (TIF) [file pone.0191444.s004.tif]

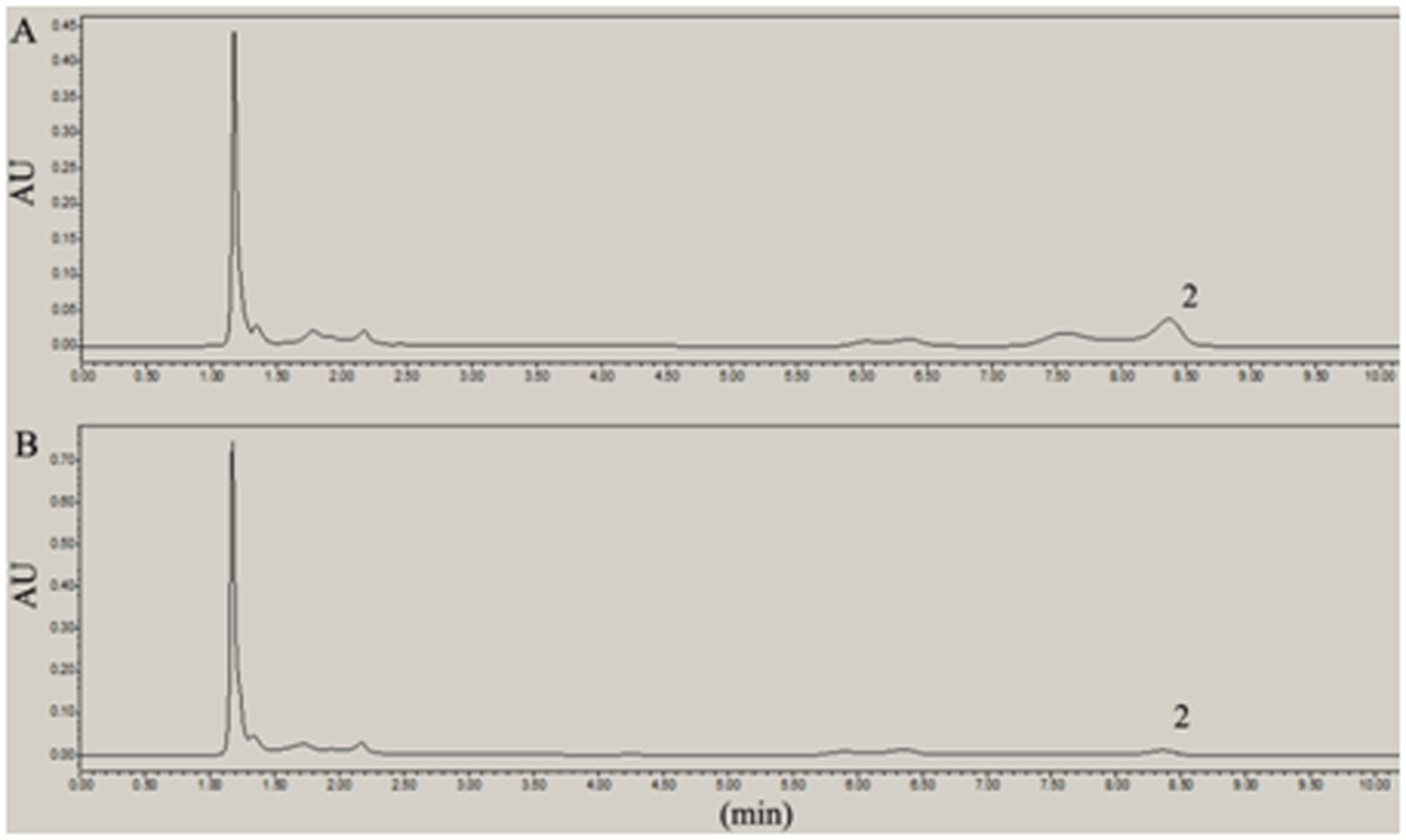

Supplement: S5 Fig — A, dark treatment; B, blue light treatment; 2, rutin. (TIF) [file pone.0191444.s005.tif]
