# Supplementary material for: Effects of blue light on flavonoid accumulation linked to the expression of miR393, miR394 and miR395 in longan embryogenic calli
Source: PLoS One. 2018 Jan 30;13(1):e0191444. doi: 10.1371/journal.pone.0191444 (PMC5790225; doi:10.1371/journal.pone.0191444)
Supplement: S1 Table — (DOCX) [file pone.0191444.s006.docx]

| **S1 Table Primers information used for real-time PCR analysis of longan genes** | | | |
| --- | --- | --- | --- |
| **Gene name** | **Primer sequences（5’→3’）** | **Size（bp）** | **TM（°C）** |
| *DlCHS*-QF | GCTTCACACAGCAATCCAGA |  |  |
|  |  | **203** | **60** |
| *DlCHS*-QR | GGATGAACAGCCCAGAACAT |  |  |
|  |  |  |  |
| *DlCHI*-QF | CAAGACTGCCGAGGAGTTGA |  |  |
|  |  | **202** | **62** |
| *DlCHI*-QR | TGAACTTCTCAGTGGCTCTGG |  |  |
|  |  |  |  |
| *DlFLS*-QF | AATGCTTCTCTCATCCTTGGC |  |  |
|  |  | **256** | **60** |
| *DlFLS*-QR | CAATCCCAATGTGAGTTCAGG |  |  |
|  |  |  |  |
| *DlF3’H*-QF | AAGAGATTGGATGCGTTCTACG |  |  |
|  |  | **230** | **62** |
| *DlF3’H*-QR | GACCGTGCTTGATGTTGTTTC |  |  |
|  |  |  |  |
| *DlDFR*-QF | CTTGTGGTTGGTCCGTTTCTA |  |  |
|  |  | **216** | **62** |
| *DlDFR*-QR | CAAGTTCAAGAATGGTGGCTG |  |  |
|  |  |  |  |
| *DlLAR*-QF | GCTGCTTTCACAATAAGTGCG |  |  |
|  |  | **117** | **62** |
| *DlLAR*-QR | TCTTACTCTCCCAGGCATCAAC |  |  |
|  |  |  |  |
| *DlTIR1-3*-QF | TGAGTAGGCAGAGAGTGTTCGT |  |  |
|  |  | **132** | **60** |
| *DlTIR1-3*-QR | GTGGGACCAAGTTGAAATCAG |  |  |
|  |  |  |  |
| *ALMT12-QF* | CCAAGGCACTCCAGACATTG |  |  |
|  |  | **107** | **62** |
| *ALMT12-QR* | CAAACATCCGTGTAGAGCAACA |  |  |
|  |  |  |  |
| *APS1-QF* | GTTGACTCCGACGATAACACG |  |  |
|  |  | **259** | **60** |
| *APS1-QR* | CACCTCCAATCAGCCAGTTTC |  |  |
|  |  |  |  |
| miR393 | CCAAAGGGATCGCATTGAT |  | **60** |
|  |  |  |  |
| miR394 | TTGGCATTCTGTCCACCTCC |  | **62** |
|  |  |  |  |
| miR395 | TTCCCCAGAACACTTCATTGG |  | **62** |
|  |  |  |  |
