# Supplementary material for: Effects of blue light on flavonoid accumulation linked to the expression of miR393, miR394 and miR395 in longan embryogenic calli
Source: PLoS One. 2018 Jan 30;13(1):e0191444. doi: 10.1371/journal.pone.0191444 (PMC5790225; doi:10.1371/journal.pone.0191444)
Supplement: S2 Table — (DOCX) [file pone.0191444.s007.docx]

| **S2 Table Growth rate of each bottle of Longan ECs on the 25 days under different light qualities** | | | | | | | | | |
| --- | --- | --- | --- | --- | --- | --- | --- | --- | --- |
| Light quality | Light intensity (µmol•m^-2^•s^-1^) | Photoperiod (h) | Every bottle growth rate 1 (%) | Every bottle growth rate 2 (%) | Every bottle growth rate 3 (%) | Average every bottle growth rate (%) | Standard deviation | Duncan (5%) | Duncan (1%) |
| Dark | 0 |  | 1045.50 | 967.00 | 1032.50 | 1015.00 | 42.074 | b | B |
| Blue | 32 | 12 | 1143.50 | 1055.25 | 1123.75 | 1107.50 | 46.315 | c | C |
| Green | 32 | 12 | 1125.25 | 1154.25 | 1110.50 | 1130.00 | 22.258 | c | C |
| White | 32 | 12 | 1012.50 | 980.25 | 962.25 | 985.00 | 25.460 | b | B |
| Red | 32 | 12 | 817.75 | 849.50 | 777.75 | 815.00 | 35.954 | a | A |
