# Supplementary material for: Effects of blue light on flavonoid accumulation linked to the expression of miR393, miR394 and miR395 in longan embryogenic calli
Source: PLoS One. 2018 Jan 30;13(1):e0191444. doi: 10.1371/journal.pone.0191444 (PMC5790225; doi:10.1371/journal.pone.0191444)
Supplement: S3 Table — (DOCX) [file pone.0191444.s008.docx]

| **S3 Table Growth rate of each bottle of Longan ECs on the 25 days under blue light of different intensities** | | | | | | | | | |
| --- | --- | --- | --- | --- | --- | --- | --- | --- | --- |
| Light quality | Light intensity (µmol•m^-2^•s^-1^) | Photoperiod (h) | Every bottle growth rate 1 (%) | Every bottle growth rate 2 (%) | Every bottle growth rate 3 (%) | Average every bottle growth rate (g FW） | Standard deviation | Duncan (5%) | Duncan (1%) |
| Dark | 0 |  | 1071.75 | 1070.25 | 1128.00 | 1090.00 | 32.918 | c | C |
| Blue | 16 | 12 | 1205.75 | 1114.25 | 1152.50 | 1157.50 | 45.954 | c | C |
| Blue | 32 | 12 | 1140.25 | 1203.25 | 1219.00 | 1187.50 | 41.671 | d | C |
| Blue | 64 | 12 | 1110.00 | 1077.50 | 1142.50 | 1110.00 | 32.500 | c | C |
| Blue | 128 | 12 | 802.00 | 862.25 | 780.75 | 815.00 | 42.277 | b | B |
| Blue | 256 | 12 | 514.75 | 585.00 | 500.25 | 533.33 | 45.328 | a | A |
