# Supplementary material for: Effects of blue light on flavonoid accumulation linked to the expression of miR393, miR394 and miR395 in longan embryogenic calli
Source: PLoS One. 2018 Jan 30;13(1):e0191444. doi: 10.1371/journal.pone.0191444 (PMC5790225; doi:10.1371/journal.pone.0191444)
Supplement: S4 Table — (DOCX) [file pone.0191444.s009.docx]

| **S4 Table Growth rate of each bottle of Longan ECs on the 25 days under blue light of different photoperiods** | | | | | | | | | |
| --- | --- | --- | --- | --- | --- | --- | --- | --- | --- |
| Light quality | Light intensity (µmol•m^-2^•s^-1^) | Photoperiod (h) | Every bottle growth rate 1 (%) | Every bottle growth rate 2 (%) | Every bottle growth rate 3 (%) | Average every bottle growth rate (%) | Standard deviation | Duncan (5%) | Duncan (1%) |
| Dark | 0 |  | 1056.75 | 1004.50 | 1058.75 | 1040.00 | 30.760 | c | C |
| Blue | 32 | 8 | 1087.50 | 1170.00 | 1187.50 | 1148.33 | 53.405 | d | D |
| Blue | 32 | 12 | 1153.75 | 1202.50 | 1206.25 | 1187.50 | 29.288 | d | D |
| Blue | 32 | 16 | 1165.25 | 1154.50 | 1130.25 | 1150.00 | 17.929 | d | D |
| Blue | 32 | 20 | 787.25 | 805.25 | 840.00 | 810.83 | 26.815 | b | B |
| Blue | 32 | 24 | 570.50 | 503.25 | 538.75 | 537.50 | 33.642 | a | A |
