# Supplementary material for: Effects of blue light on flavonoid accumulation linked to the expression of miR393, miR394 and miR395 in longan embryogenic calli
Source: PLoS One. 2018 Jan 30;13(1):e0191444. doi: 10.1371/journal.pone.0191444 (PMC5790225; doi:10.1371/journal.pone.0191444)
Supplement: S5 Table — (DOCX) [file pone.0191444.s010.docx]

| **S5 Table Flavonoid contents of longan ECs under blue light of different qualities** | | | | | | | | |  |
| --- | --- | --- | --- | --- | --- | --- | --- | --- | --- |
| Light quality | Light intensity (µmol•m^-2^•s^-1^) | Photoperiod (h) | Flavonoids content 1 (mg/g DW) | Flavonoids content 2 (mg/g DW) | Flavonoids content 3 (mg/g DW) | Average flavonoids content (mg/g DW) | Standard deviation | Duncan (5%) | Duncan (1%) |
| Dark | 0 |  | 10.307 | 10.872 | 10.42 | 10.533 | 0.299 | a | A |
| Blue | 32 | 12 | 17.77 | 18.165 | 17.374 | 17.770 | 0.396 | d | D |
| Green | 32 | 12 | 16.808 | 16.356 | 16.582 | 16.582 | 0.226 | c | C |
| White | 32 | 12 | 12.512 | 12.964 | 12.738 | 12.738 | 0.226 | b | B |
| Red | 32 | 12 | 10.024 | 10.476 | 10.194 | 10.231 | 0.228 | a | A |
