# Supplementary material for: Effects of blue light on flavonoid accumulation linked to the expression of miR393, miR394 and miR395 in longan embryogenic calli
Source: PLoS One. 2018 Jan 30;13(1):e0191444. doi: 10.1371/journal.pone.0191444 (PMC5790225; doi:10.1371/journal.pone.0191444)
Supplement: S6 Table — (DOCX) [file pone.0191444.s011.docx]

| **S6 Table Epicatechin contents of longan ECs under different treatments** | | | | | | | | |  |
| --- | --- | --- | --- | --- | --- | --- | --- | --- | --- |
| Light quality | Light intensity (µmol•m^-2^•s^-1^) | Photoperiod (h) | Epicatechin content 1 (ug/g DW) | Epicatechin content 2 (ug/g DW) | Epicatechin content 3 (ug/g DW) | Average epicatechin content (ug/g DW) | Standard deviation | Duncan (5%) | Duncan (1%) |
| Dark | 0 |  | 658.10 | 639.21 | 670.12 | 655.81 | 15.582 | a | a |
| Blue | 32 | 12 | 2219.17 | 2232.30 | 2180.52 | 2210.66 | 26.918 | b | b |
