# Supplementary material for: Effects of blue light on flavonoid accumulation linked to the expression of miR393, miR394 and miR395 in longan embryogenic calli
Source: PLoS One. 2018 Jan 30;13(1):e0191444. doi: 10.1371/journal.pone.0191444 (PMC5790225; doi:10.1371/journal.pone.0191444)
Supplement: S7 Table — (DOCX) [file pone.0191444.s012.docx]

| **S7 Table Rutin contents of longan ECs under different treatments** | | | | | | | | |  |
| --- | --- | --- | --- | --- | --- | --- | --- | --- | --- |
| Light quality | Light intensity (µmol•m^-2^•s^-1^) | Photoperiod (h) | Rutin content 1 (ug/g DW) | Rutin content 2 (ug/g DW) | Rutin content 3 (ug/g DW) | Average rutin content (ug/g DW) | Standard deviation | Duncan (5%) | Duncan (1%) |
| Dark | 0 |  | 1809.36 | 1850.32 | 1790.45 | 1816.71 | 30.604 | b | b |
| Blue | 32 | 12 | 161.42 | 165.43 | 162.5 | 163.12 | 2.075 | a | a |
