# Supplementary material for: Effects of blue light on flavonoid accumulation linked to the expression of miR393, miR394 and miR395 in longan embryogenic calli
Source: PLoS One. 2018 Jan 30;13(1):e0191444. doi: 10.1371/journal.pone.0191444 (PMC5790225; doi:10.1371/journal.pone.0191444)
Supplement: S8 Table — (DOCX) [file pone.0191444.s013.docx]

| **S8 Table Flavonoid contents of longan ECs under blue light of different intensities** | | | | | | | | | | |  |
| --- | --- | --- | --- | --- | --- | --- | --- | --- | --- | --- | --- |
| Light quality | Light intensity (µmol•m^-2^•s^-1^) | Photoperiod (h) | Flavonoids content 1 (mg/g DW) | Flavonoids content 2 (mg/g DW) | Flavonoids content 3 (mg/g DW) | | Average flavonoids content (mg/g DW) | | Standard deviation | Duncan (5%) | Duncan (1%) |
| Dark | 0 |  | 9.741 | 9.289 | 9.176 | 9.40 | | 0.299 | | a | A |
| Blue | 16 | 12 | 16.300 | 17.317 | 16.808 | 16.81 | | 0.509 | | d | D |
| Blue | 32 | 12 | 18.448 | 18.392 | 19.466 | 18.77 | | 0.605 | | e | E |
| Blue | 64 | 12 | 13.699 | 13.982 | 13.755 | 13.81 | | 0.150 | | c | C |
| Blue | 128 | 12 | 12.286 | 12.964 | 12.286 | 12.51 | | 0.392 | | b | B |
| Blue | 256 | 12 | 17.430 | 17.261 | 17.374 | 17.36 | | 0.086 | | d | D |
