# Supplementary material for: Effects of blue light on flavonoid accumulation linked to the expression of miR393, miR394 and miR395 in longan embryogenic calli
Source: PLoS One. 2018 Jan 30;13(1):e0191444. doi: 10.1371/journal.pone.0191444 (PMC5790225; doi:10.1371/journal.pone.0191444)
Supplement: S9 Table — (DOCX) [file pone.0191444.s014.docx]

| **S9 Table Flavonoid contents of longan ECs under blue light of different photoperiods** | | | | | | | | |  |
| --- | --- | --- | --- | --- | --- | --- | --- | --- | --- |
| Light quality | Light intensity (µmol•m^-2^•s^-1^) | Photoperiod (h) | Flavonoids content 1 (mg/g DW) | Flavonoids content 2 (mg/g DW) | Flavonoids content 3 (mg/g DW) | Average flavonoids content (mg/g DW) | Standard deviation | Duncan (5%) | Duncan (1%) |
| Dark | 0 |  | 8.400 | 8.441 | 8.384 | 8.41 | 0.029 | a | A |
| Blue | 32 | 8 | 14.434 | 14.886 | 14.999 | 14.77 | 0.299 | e | D |
| Blue | 32 | 12 | 16.695 | 16.582 | 17.148 | 16.81 | 0.299 | f | E |
| Blue | 32 | 16 | 11.777 | 12.003 | 11.494 | 11.76 | 0.255 | b | B |
| Blue | 32 | 20 | 12.399 | 12.229 | 12.738 | 12.46 | 0.259 | c | C |
| Blue | 32 | 24 | 14.377 | 14.547 | 14.038 | 14.32 | 0.259 | d | D |
