# Supplementary material for: Effects of blue light on flavonoid accumulation linked to the expression of miR393, miR394 and miR395 in longan embryogenic calli
Source: PLoS One. 2018 Jan 30;13(1):e0191444. doi: 10.1371/journal.pone.0191444 (PMC5790225; doi:10.1371/journal.pone.0191444)
Supplement: S11 Table — (DOCX) [file pone.0191444.s016.docx]

| **S11 Table The expression of flavonoid metabolic pathway genes of longan ECs under blue light of different photoperiods** | | | | | | | | | | | | | | |
| --- | --- | --- | --- | --- | --- | --- | --- | --- | --- | --- | --- | --- | --- | --- |
| Light quality | Light intensity (µmol•m^-2^•s^-1^) | Photoperiod (h) | DlCHS | | DlCHI | | DlFLS | | DlF3'H | | DlDFR | | DlLAR | |
|  |  |  | Relative expression | SD | Relative expression | SD | Relative expression | SD | Relative expression | SD | Relative expression | SD | Relative expression | SD |
| Dark | 0 |  | 0.37 | 0.04 | 0.17 | 0.03 | 1.16 | 0.11 | 0.62 | 0.08 | 0.09 | 0.01 | 0.48 | 0.05 |
| Blue | 32 | 8 | 0.59 | 0.07 | 0.40 | 0.06 | 0.87 | 0.11 | 0.22 | 0.08 | 0.33 | 0.05 | 1.08 | 0.12 |
| Blue | 32 | 12 | 0.74 | 0.08 | 1.53 | 0.12 | 0.42 | 0.05 | 0.64 | 0.05 | 0.75 | 0.14 | 0.90 | 0.11 |
| Blue | 32 | 16 | 0.54 | 0.05 | 0.30 | 0.03 | 0.64 | 0.08 | 0.33 | 0.11 | 0.28 | 0.07 | 0.75 | 0.12 |
| Blue | 32 | 20 | 0.75 | 0.12 | 1.81 | 0.20 | 0.51 | 0.06 | 2.17 | 0.23 | 0.32 | 0.07 | 1.25 | 0.14 |
| Blue | 32 | 24 | 1.81 | 0.16 | 0.55 | 0.13 | 0.50 | 0.04 | 0.24 | 0.04 | 1.81 | 0.13 | 1.81 | 0.13 |
