# Supplementary material for: Effects of blue light on flavonoid accumulation linked to the expression of miR393, miR394 and miR395 in longan embryogenic calli
Source: PLoS One. 2018 Jan 30;13(1):e0191444. doi: 10.1371/journal.pone.0191444 (PMC5790225; doi:10.1371/journal.pone.0191444)
Supplement: S12 Table — (DOCX) [file pone.0191444.s017.docx]

**S12 Table The expression of miRNAs and flavonoid metabolish related genes of longan ECs under blue light**

| Light quality | Light intensity (µmol•m^-2^•s^-1^) | Photoperiod (h) | DlCHS | | | DlCHI | | | DlFLS | | | DlF3'H | | |
| --- | --- | --- | --- | --- | --- | --- | --- | --- | --- | --- | --- | --- | --- | --- |
|  |  |  | Relative expression | SD | Duncan (1%) | Relative expression | SD | Duncan (1%) | Relative expression | SD | Duncan (1%) | Relative expression | SD | Duncan (1%) |
| Dark | 0 |  | 0.34 | 0.040 | a | 0.50 | 0.046 | a | 1.61 | 0.046 | b | 0.74 | 0.067 | a |
| Blue | 32 | 12 | 1.00 | 0.092 | b | 1.00 | 0.077 | b | 1.00 | 0.077 | a | 1.00 | 0.066 | b |

**S12 Table The expression of miRNAs and flavonoid metabolish related genes of longan ECs under blue light (continued)**

| Light quality | Light intensity (µmol•m^-2^•s^-1^) | Photoperiod (h) | DlDFR | | | DlLAR | | | DlTIR1-3 | | | DlALMT12 | | |
| --- | --- | --- | --- | --- | --- | --- | --- | --- | --- | --- | --- | --- | --- | --- |
|  |  |  | Relative expression | SD | Duncan (1%) | Relative expression | SD | Duncan (1%) | Relative expression | SD | Duncan (1%) | Relative expression | SD | Duncan (1%) |
| Dark | 0 |  | 0.45 | 0.032 | a | 0.71 | 0.068 | a | 0.70 | 0.070 | a | 0.57 | 0.051 | a |
| Blue | 32 | 12 | 1.00 | 0.072 | b | 1.00 | 0.071 | b | 1.00 | 0.075 | b | 1.00 | 0.077 | b |

**S12 Table The expression of miRNAs and flavonoid metabolish related genes of longan ECs under blue light (continued)**

| Light quality | Light intensity (µmol•m^-2^•s^-1^) | Photoperiod (h) | DlAPS1 | | | miR393 | | | miR394 | | | miR395 | | |
| --- | --- | --- | --- | --- | --- | --- | --- | --- | --- | --- | --- | --- | --- | --- |
|  |  |  | Relative expression | SD | Duncan (1%) | Relative expression | SD | Duncan (1%) | Relative expression | SD | Duncan (1%) | Relative expression | SD | Duncan (1%) |
| Dark | 0 |  | 0.69 | 0.056 | a | 1.15 | 0.084 | b | 1.15 | 0.068 | b | 1.15 | 0.073 | b |
| Blue | 32 | 12 | 1.00 | 0.087 | b | 0.84 | 0.097 | a | 0.89 | 0.078 | a | 0.53 | 0.046 | a |
