# Supplementary material for: Effects of blue light on flavonoid accumulation linked to the expression of miR393, miR394 and miR395 in longan embryogenic calli
Source: PLoS One. 2018 Jan 30;13(1):e0191444. doi: 10.1371/journal.pone.0191444 (PMC5790225; doi:10.1371/journal.pone.0191444)
Supplement: S13 Table — (DOCX) [file pone.0191444.s018.docx]

| **S13 Table The expression of miRNAs and flavonoid metabolish related genes under blue light of different intensities** | | | | | | | | | | | | | | |
| --- | --- | --- | --- | --- | --- | --- | --- | --- | --- | --- | --- | --- | --- | --- |
| Light quality | Light intensity (µmol•m^-2^•s^-1^) | Photoperiod (h) | DlCHS | | DlCHI | | DlFLS | | DlF3'H | | DlDFR | | DlLAR | |
|  |  |  | Relative expression | SD | Relative expression | SD | Relative expression | SD | Relative expression | SD | Relative expression | SD | Relative expression | SD |
| Dark | 0 |  | 1.00 | 0.09 | 1.00 | 0.08 | 1.00 | 0.08 | 1.00 | 0.42 | 1.00 | 0.20 | 1.00 | 0.14 |
| Blue | 16 | 12 | 3.64 | 0.42 | 3.34 | 0.28 | 0.71 | 0.05 | 4.80 | 0.31 | 6.23 | 0.27 | 9.69 | 0.56 |
| Blue | 32 | 12 | 8.03 | 0.53 | 8.07 | 0.46 | 0.65 | 0.05 | 8.17 | 0.15 | 8.18 | 0.31 | 8.03 | 0.41 |
| Blue | 64 | 12 | 7.49 | 0.38 | 4.93 | 0.64 | 0.45 | 0.03 | 8.91 | 0.78 | 2.08 | 0.32 | 7.33 | 0.47 |
| Blue | 128 | 12 | 3.31 | 0.38 | 4.34 | 0.22 | 0.49 | 0.02 | 3.07 | 0.31 | 0.39 | 0.05 | 4.95 | 0.26 |
| Blue | 256 | 12 | 3.90 | 0.47 | 3.26 | 0.32 | 0.70 | 0.05 | 5.35 | 0.86 | 1.68 | 0.30 | 4.40 | 0.21 |

**S13 Table The expression of miRNAs and flavonoid metabolish related genes under blue light of different intensities** **(continued)**

| Light quality | Light intensity (µmol•m^-2^•s^-1^) | Photoperiod (h) | DlTIR1-3 | | DlALMT12 | | DlAPS1 | | miR393 | | miR394 | | miR395 | |
| --- | --- | --- | --- | --- | --- | --- | --- | --- | --- | --- | --- | --- | --- | --- |
|  |  |  | Relative expression | SD | Relative expression | SD | Relative expression | SD | Relative expression | SD | Relative expression | SD | Relative expression | SD |
| Dark | 0 |  | 1.00 | 0.08 | 0.85 | 0.21 | 0.46 | 0.03 | 3.76 | 0.47 | 4.30 | 0.38 | 1.34 | 0.18 |
| Blue | 16 | 12 | 5.81 | 0.38 | 5.62 | 0.73 | 3.97 | 0.36 | 0.80 | 0.20 | 0.41 | 0.04 | 0.55 | 0.06 |
| Blue | 32 | 12 | 6.93 | 0.36 | 3.65 | 0.48 | 2.18 | 0.23 | 2.96 | 0.43 | 0.99 | 0.27 | 0.71 | 0.20 |
| Blue | 64 | 12 | 3.63 | 0.22 | 4.49 | 0.51 | 15.32 | 1.03 | 6.80 | 0.28 | 2.04 | 0.26 | 4.81 | 0.60 |
| Blue | 128 | 12 | 4.45 | 0.25 | 2.49 | 0.19 | 3.45 | 0.33 | 8.59 | 0.52 | 1.97 | 0.34 | 5.21 | 0.41 |
| Blue | 256 | 12 | 5.15 | 0.43 | 9.74 | 0.65 | 7.06 | 0.74 | 4.22 | 0.57 | 0.55 | 0.06 | 2.24 | 0.31 |
